# Transcriptomic Analysis Suggests Auxin Regulation in Dorsal-Ventral Petal Asymmetry of Wild Progenitor *Sinningia speciosa*

**DOI:** 10.3390/ijms23042073

**Published:** 2022-02-13

**Authors:** Zhao-Jun Pan, Ya-Chi Nien, Yu-An Shih, Tsun-Ying Chen, Wen-Dar Lin, Wen-Hsi Kuo, Hao-Chun Hsu, Shih-Long Tu, Jen-Chih Chen, Chun-Neng Wang

**Affiliations:** 1 Department of Life Science, National Taiwan University, Taipei 10617, Taiwan; zoeypan00@gmail.com (Z.-J.P.); yxn5072@psu.edu (Y.-C.N.); r04b42012@ntu.edu.tw (Y.-A.S.); andy870325@gmail.com (T.-Y.C.); 2 Institute of Plant and Microbial Biology, Academia Sinica, Taipei 11529, Taiwan; wdlin@gate.sinica.edu.tw (W.-D.L.); tsl@gate.sinica.edu.tw (S.-L.T.); 3 Institute of Ecology and Evolutionary Biology, National Taiwan University, Taipei 10617, Taiwan; kuowenhsi@gmail.com (W.-H.K.); kent.haochunhsu@gmail.com (H.-C.H.); 4 Institute of Biotechnology, National Taiwan University, Taipei 10617, Taiwan

**Keywords:** auxin, cell growth, floral symmetry, petal pigmentation, petal size

## Abstract

The establishment of dorsal–ventral (DV) petal asymmetry is accompanied by differential growth of DV petal size, shape, and color differences, which enhance ornamental values. Genes involved in flower symmetry in *Sinningia speciosa* have been identified as *CYCLOIDEA* (*SsCYC*), but which gene regulatory network (GRN) is associated with *SsCYC* to establish DV petal asymmetry is still unknown. To uncover the GRN of DV petal asymmetry, we identified 630 DV differentially expressed genes (DV-DEGs) from the RNA-Seq of dorsal and ventral petals in the wild progenitor, *S. speciosa* ‘ES’. Validated by qRT-PCR, genes in the auxin signaling transduction pathway, *SsCYC*, and a major regulator of anthocyanin biosynthesis were upregulated in dorsal petals. These genes correlated with a higher endogenous auxin level in dorsal petals, with longer tube length growth through cell expansion and a purple dorsal color. Over-expression of *SsCYC* in *Nicotiana* reduced petal size by regulating cell growth, suggesting that *SsCYC* also controls cell expansion. This suggests that auxin and *SsCYC* both regulate DV petal asymmetry. Transiently over-expressed *SsCYC*, however, could not activate most major auxin signaling genes, suggesting that *SsCYC* may not trigger auxin regulation. Whether auxin can activate *SsCYC* or whether they act independently to regulate DV petal asymmetry remains to be explored in the future.

## 1. Introduction

Floral zygomorphy (bilateral symmetry, dorsal–ventral asymmetry) has been shown to be a key innovation that is often associated with flower diversity in many angiosperms due to its role in facilitating plant–pollinator interactions. Zygomorphy has evolved independently in many angiosperm lineages [1]. Within the Lamiales, the flowers of many outcrossing zygomorphic lineages in the Gesneriaceae show diversification in size, shape, and color that are associated with pollination specializations [2,3,4]. Species in the genus *Sinningia* (subfamily Gesnerioideae, tribe Gesnerieae) are remarkably diverse, with flowers that have features consistent with bee, hummingbird, bat, and moth pollination syndromes [5]. Floral morphologies, composed of various tubular shapes and pigment patterns, are shown to be correlated with different types of pollination syndromes, and further affect flower diversification in Gesneriaceae [5,6,7]. The development of floral dorsal–ventral asymmetry therefore appears to be associated with flower pollination traits relating to size, shape, and color.

**Figure 1:**
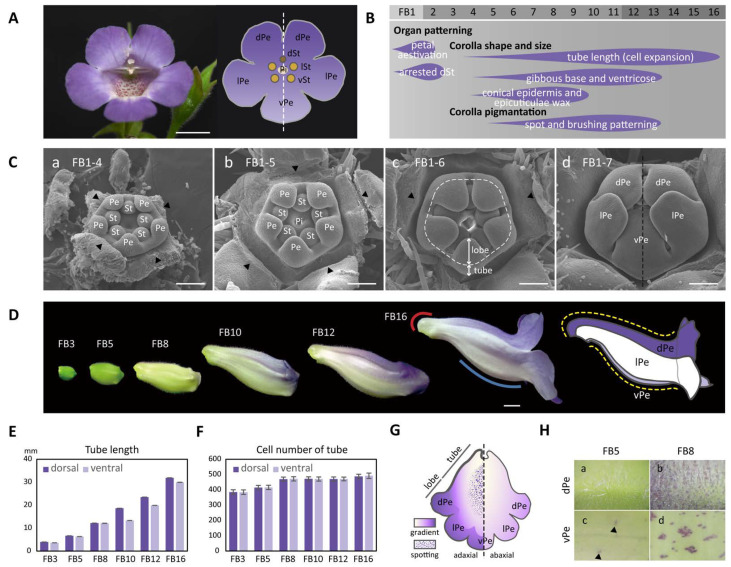
The establishment of dorsal–ventral petal asymmetry in shape, size, and pigmentation patterns in *S. speciosa* ‘ES’. (**A**) A front view of the fully opened zygomorphic flower (left) and the floral diagram (right). Scale bars = 10 mm. (**B**) The flower bud developmental stages marked with features of the asymmetric dorsal–ventral petal growth relating to corolla shape, size, and pigmentation. (**C**) SEM of floral organ primordia development along sub-stages (1-4~1-7) of floral bud stage 1 (FB1). Scale bars = 100 μm. (**a**–**c**) Petal and stamen primordia are equal in size (actinomorphy) during sub-stages FB1-4 to FB1-6. Arrowheads indicate the removed sepals and the dashed line in (**c**) marks the boundary between the corolla tube and petal lobes. (**d**) Initiated petal zygomorphy at FB1-7. (**D**) Corolla tube development from stages FB3 to FB16, during which the dorsal–ventral petal asymmetry is established gradually. The red and blue lines indicate a gibbous outgrowth at the base of the dorsal corolla tube (red curve), and a ventricose chamber (blue curve) at the ventral corolla tube. Refer to the main text for a detailed explanation. The dashed yellow lines demonstrate the tube length difference along the curvature of the dorsal and ventral corolla tubes. Scale bars = 5 mm. (**E**,**F**) The measurement of tube length (**E**) and cell number (**F**) between the dorsal and ventral parts of the corolla. (**G**) A cartoon illustration showing the gradient and spotting pigmentation patterns on the adaxial and abaxial surfaces of the corolla tube at FB16. (**H**) Purple pigments (anthocyanin) start to accumulate on cells of the adaxial surface of the dorsal petal at FB5 and become evident at FB8. (**a**,**b**) Purple spots also appear on the adaxial surface of the ventral petal at FB5 (arrowheads) but become obvious at FB8 (**c**,**d**). dPe, dorsal petal; lPe, lateral petal; vPe, ventral petal; dSt, dorsal staminode; lSt, lateral stamen; vSt, ventral stamen; Pi, pistil; Pe, petal; St, stamen primordia.

*Sinningia speciosa* (Lodd.) Hiern ‘Espírito Santo’ (‘ES’) is a bee-pollinated wild perennial species. The zygomorphic corolla tube of *S. speciosa* is distinguished by an asymmetric dorsal–ventral petal contour that comprises prominent ventral swelling and dorsal gibbosity (nectar chamber) at the base of the corolla tube (Figure 1). In addition, the corolla exhibits an asymmetrical dorsal–ventral purple pigmentation pattern, in which dark spots or stripes are present on the inner surface of the ventral corolla tube, which likely serve as a nectar guide for visiting insects. These pollination-related traits such as dorsal–ventral petal size, petal shape, and pigmentation differences in the corolla therefore appear to correlate with the development of floral zygomorphy of *S. speciosa*.

The key genes responsible for establishing the dorsal–ventral identity of flowers have been shown to be homologs of *CYCLOIDEA* (*CYC*) of *Antirrhinum majus* in Lamiales and almost all major angiosperm lineages [8,9]. *CYC* and its paralog *DICHOTOMA* (*DICH*) activate their downstream target MYB gene, *RADIALIS* (*RAD*), in dorsal petals by directly binding to the *RAD* promoter [10]. In contrast, the ventral identity is established by a MYB protein complex consisting of DIVARICATA (DIV) and DIV-and-RAD-interacting-factor (DRIF), which controls the expression of downstream genes involved in floral ventralization. In dorsal petals, the RAD protein can bind to DRIF and prevent the formation of the DIV/DRIF complex [11]. Therefore, the ventralizing effect of *DIV* is restricted to the ventral petals, while *CYC* activates *RAD* in the dorsal petals, thus creating the dorsal–ventral polarity. In Gesneriaceae species, diversified *CYC* expression patterns have been found to correlate with floral symmetry transition and flower shape diversity [12,13,14]. However, little is known about which gene regulatory network (GRN) is necessary in addition to *CYC-RAD* and *DIV* in generating the size and shape differences between dorsal and ventral petals.

*CYC*-like genes belong to the *TCP* gene family encoding plant-specific proteins sharing a basic helix–loop–helix (bHLH) motif, the so-called TCP domain that allows DNA binding and regulates plant growth and development [15]. Several functional studies have shown that *CYC*-like genes in the class II TCP family are involved in regulating cell proliferation or expansion to affect organ size. Transgenic plants of *Arabidopsis thaliana* over-expressing the *CYC* gene from *A. majus* show increased petal size as a consequence of an increase in cell expansion in petals, whereas *CYC* reduces both cell proliferation (by promoting the early arrest of cell division) and cell expansion in leaves [16,17]. Among Gesneriaceae species, over-expressing the *SiCYC1A* gene from the African violet (*Streptocarpus ionanthus*) in *A. thaliana* results in a reduction in petal size due to decreased cell proliferation [14]. The constitutive expression of *CYC1C* homologous genes from *Primulina heterotricha* and *SsCYC* from *S*. *speciosa* in *A. thaliana*, however, reduce petal size via the regulation of decreased cell growth [18,19]. Thus, there appear to be no simple rule to predict the effects of *CYC* homologs on petal growth across angiosperm lineages.

Although flowers of wild progenies of *S. speciosa* are zygomorphic, many actinomorphic flower cultivars have been favored and selected by humans, attributing to a *SsCYC* coding region deletion [19]. Efforts in crossing the zygomorphic wildtype (containing functional *SsCYC* alleles) with the actinomorphic peloria (containing mutated *Sscyc* alleles) resulted in the F1 generation, which are all zygomorphic [19,20,21]. The F2 generation from F1 selfing are segregated at a 3:1 ratio of dominancy of zygomorphy to actinomorphy, and this allows genotype–phenotype associations of *SsCYC* alleles to petal morphology [20,21]. Geometric morphometric analysis of 3D petal morphology of *S. speciosa* has been shown to be useful to analyze flower shape and size variation [20,22]. The associations of *SsCYC* alleles with 3D petal morphology reveal that the functional *SsCYC* allele correlates with dorsal petal outward curvature (e.g., the gibbous structure—nectar chamber) and ventral outward curvature (e.g., the ventricose chamber in the ventral tube, see our Figure 1) [21]. This means that *SsCYC* not only functions to establish flower dorsal–ventral identity, but may also affect petal shape, size, and color differences between dorsal and ventral petals.

We therefore would like to identify what developmental modules, in addition to *SsCYC*, could contribute to dorsal–ventral petal shape and color asymmetry in the wild zygomorphic progeny of *Sinningia speciosa* ‘ES’. We hypothesize that the gene regulatory networks involved in regulating cell growth and the purple color gradient between the dorsal and ventral petals are likely accompanied by the establishment of floral symmetry. There is currently little information about what GRN may be dependent or independent of *SsCYC* in creating dorsal–ventral petal asymmetry across angiosperms. In this study, we therefore performed RNA-Seq separately on the dorsal and ventral petals during a single floral bud developmental stage, establishing floral zygomorphy when *SsCYC* is dorsally highly expressed. We first characterized flower buds to ascertain the stages in which the major morphological changes occurred during the establishment of dorsal–ventral petal asymmetry using SEM and petal size measurement. These morphological observations helped to identify differentially expressed genes that relate to dorsal–ventral petal asymmetry. Auxin signaling pathway genes, together with the *SsCYC*-*SsRAD* regulatory module and the major regulator of anthocyanin biosynthesis, showed dorsal–ventral asymmetrical expressions. A model illustrating how the regulation of these genes correlates with asymmetric dorsal–ventral petal growth and pigmentation patterns in *S. speciosa* ‘ES’ was proposed.

## 2. Results

### 2.1. The Development of Zygomorphy Is Due to Shape and Size Differences in the Dorsal and Ventral Parts of the Corolla Tube

To characterize when the floral zygomorphy in *S. speciosa* ‘ES’ was initially established, we examined the developing floral primordia throughout floral bud initiation stages using SEM. Floral zygomorphy, however, was not established in the early bud stage until FB1-7 (ca. 0.5 mm in diameter) (Figure 1(Ca–Cc)). At stage FB1-4, each of the five petal primordia and five stamen primordia were equal in size (Figure 1(Ca)). Floral symmetry thus appears to be actinomorphic at the beginning of petal and stamen organogenesis. Later during development, at stages of FB1-5 and FB1-6, all five petal primordia continued to grow at the same rate and started to differentiate into the tube at the base and the petal lobes towards the tip (Figure 1(Cb,Cc). At stage FB1-7, while the tube further elongated, the corolla lobes enlarged to enclose the stamens and pistils. The arrangement of five lobes developed into a specified aestivation pattern showing two lateral lobes enfolding the dorsal (middle) and ventral (innermost) petal lobes (Figure 1(Cd)). The floral zygomorphy along the dorsal–ventral axis therefore initiates at FB1-7 (the dashed line in Figure 1(Cd)). The development of the aborted dorsal staminode probably also follows a dorsal–ventral asymmetry pattern as early as at FB1-8 (Figure S1A and Table S1).

**Figure 2:**
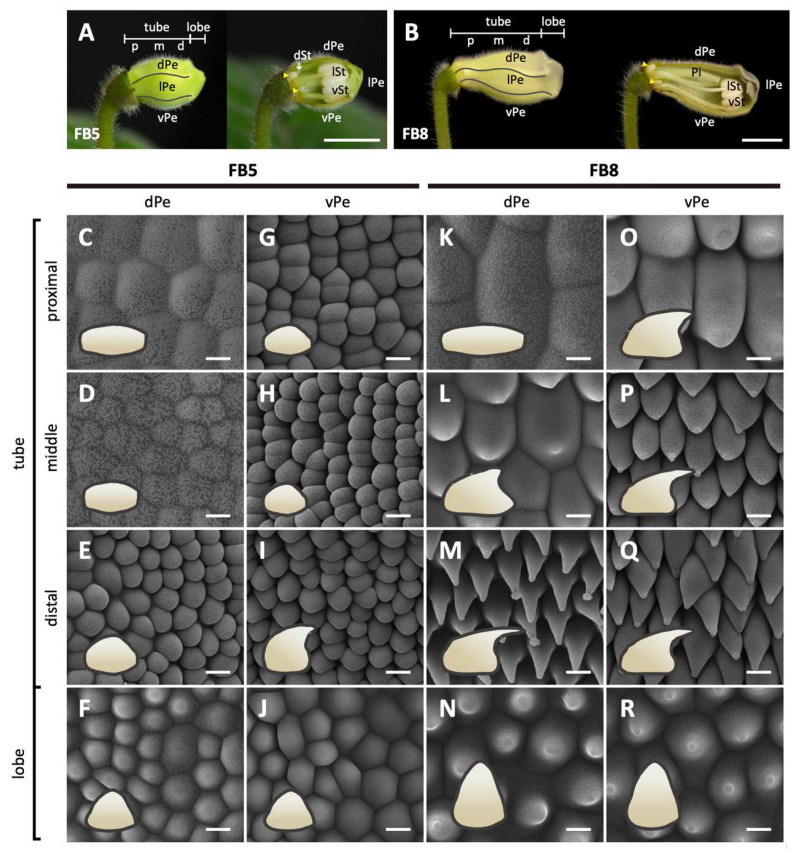
Morphological changes in the epidermal cells of the dorsal and ventral petals of *S. speciosa* ‘ES’ flowers. (**A**,**B**) The overview and longitudinal sections of floral buds at stages FB5 and FB8. The white arrow indicates the arrested growth of the dorsal staminode, and the yellow arrowheads show the lateral nectaries. Scale bars = 1 cm. (**C**–**R**) Top-view Cryo-SEM images of the adaxial epidermis of dorsal and ventral petals. Side-views of epidermal cells are illustrated as cartoon pictures in the bottom-left of each panel. Scale bars = 10 μm. dPe, dorsal petal; lPe, lateral petal; vPe, ventral petal; dSt, dorsal staminode; lSt, lateral stamen; vSt, ventral stamen; Pi, pistil. Different regions of the corolla tube: p, proximal; m, middle; d, distal.

In the later floral stages, FB3-FB16, the flower buds gradually assumed the differences in shape between the dorsal and ventral corolla parts (Figure 1D). The development of flower dorsal–ventral asymmetry was characterized by a differential degree of curvature between the dorsal and ventral parts of the corolla tube (yellow dashed curved lines). Additionally, this asymmetry was accompanied by a convex, gibbous outgrowth (nectar chamber) at the base of the dorsal corolla tube (red curve), and a ventricose chamber (blue curve) in the ventral part of the corolla tube (Figure 1D). The difference in the tube lengths between the dorsal and ventral parts of the corolla became evident starting at developmental stage FB10 (Figure 1E). The dorsal tube length gradually became longer than the ventral part of the tube during FB10 and FB12. However, the length difference between the dorsal tube and ventral tube narrowed again at FB16. Moreover, the number of tube cells in the dorsal and ventral parts remained almost equal across all flower developmental stages (Figure 1F). Thus, we can conclude that the dorsal–ventral corolla size difference was mainly due to the larger degree of cell expansion in length in the dorsal part compared to the ventral part of the corolla. We further examined surface cellular micromorphology of the petal epidermal cells with a focus on cell size and shape differences using cryo-SEM at FB5 and FB8 stages (Figure 2A,B). The dorsal part of the corolla had larger, flattened, and rougher epidermal cells (Figure 2C,D vs. Figure 2K,L), while the ventral part had relatively smaller, more protruding conical cells and a smooth epidermis, particularly in the proximal and middle regions (Figure 2G,H vs. Figure 2O,P). Moreover, there are more granule-like epicuticular wax crystals deposited on the epidermal surface of the dorsal part of the corolla tube than on the ventral part (Figure 2K–R and Figure S1B). Thus, the development of petal epidermal micromorphology in zygomorphic *S. speciosa* ‘ES’ corolla petals followed a dorsal–ventral asymmetry pattern.

### 2.2. Flower Coloration in Zygomorphic S. speciosa Displays a Distinct Dorsal–Ventral Pigmentation Pattern

The development of flower dorsal–ventral asymmetry in *S. speciosa* ‘ES’ is not only apparent in the differences in corolla shape and size, but is also evident in pigmentation pattern. The corolla begins to show its purple gradient coloration along the dorsal–ventral and proximal–distal axes at stages FB8, especially on the dorsal side, while at FB5 and earlier stages, the entire corolla is green (Figure 1D). The color becomes more intense and spreads from the dorsal tube to the dorsal lobes beginning in stage FB10 (Figure 1G). The color is a sign that purple anthocyanin has accumulated. This can be observed by the accumulation of purple pigments in corolla epidermal cells (Figure 1H). On the dorsal tube of stage FB8 flowers, a number of epidermal cells, particularly those at the tip of the trichomes, show the purple color (Figure 1(Hb)). At anthesis (stage FB16), almost all epidermal and trichome cells on the dorsal surface of the corolla highly accumulate purple pigments (Figure S1C). On the inner (adaxial) surface of the ventral tube, certain epidermal cells may also produce purple anthocyanin starting at FB5 (Figure 1(Hc)). These purple-colored epidermal cells later accumulate in clusters from stage FB8 to anthesis at FB16 (Figure 1(Hd) and Figure S1C), thus exhibiting a spotting pattern on the inner surface of the ventral tube. Therefore, the display of the ventral petal spotting pattern also follows a position-dependent pattern along the dorsal–ventral axis of the flower in *S. speciosa* ‘ES’.

### 2.3. Transcriptome Analysis of DEGs between the Dorsal and Ventral Petals

RNA-Seq data between the dorsal and ventral petals obtained from FB5 were chosen because dorsal–ventral petal asymmetry was established and high *SsCYC* expression in the dorsal part was observed (Figure S2A,B). A total of 262,599,569 clean reads from four libraries (ZD-1, ZD-2, ZV-1, and ZV-2) were obtained (Table S2) and were individually mapped to the predicted transcripts from draft genome sequences of *S. speciosa* ‘Avenida Niemeyer’ (‘AN’), which is closely related to *S. speciosa* ‘ES’. Of the clean reads, 84.5–85.3% aligned (Table S2), indicating that the draft genome sequence was an acceptable reference for our transcriptomic analysis. A total of 630 dorsal–ventral differentially expressed genes (DV-DEGs) were obtained from the transcriptomic comparison between ZDs and ZVs. The heatmap shows the relative expression levels of the 294 dorsally high and 336 ventrally high DEGs (Figure S3).

### 2.4. Functional Annotation and GO Enrichment Analysis of the Dorsal–Ventral DEGs

**Figure 3:**
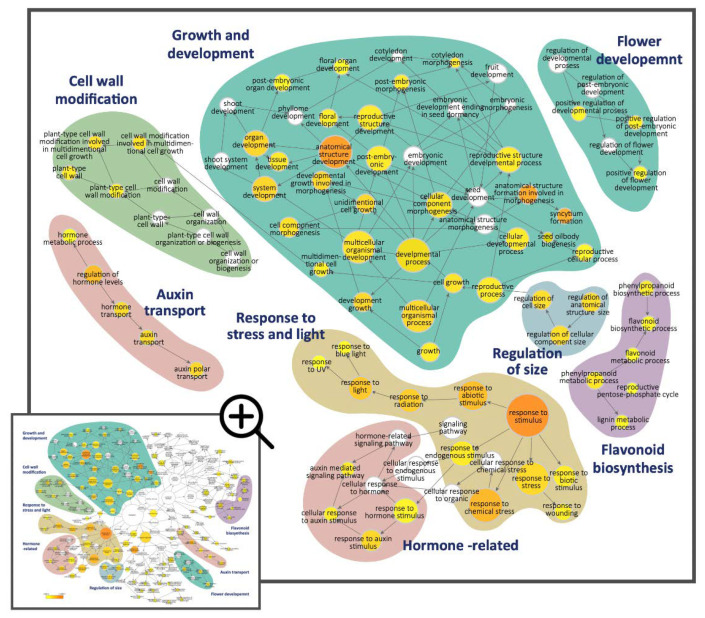
The Gene Ontology (GO) enrichment analysis for the significantly enriched GO terms belonging to the ‘biological process’ GO category for the 630 DV-DEGs in *S. speciosa* ‘ES’. Node size is proportional to the number of genes, and the color scale represents the significance levels: white, no significant difference; yellow, *p*-value < 5 × 10^−2^; orange, *p*-value < 5 × 10^−7^. Color shadings refer to the GO terms that relate to six broad biological functions. Arrows indicate connections between GO annotations.

The DV-DEGs were then functionally annotated against the GeneBank NR, GO, and KEGG public databases (Table S3). GO functional classification assigned the DV-DEGs to 796 GO terms in the three main GO categories and 39 sub-categories. GO terms in the ‘biological process’ category, including ‘biological regulation’, ‘localization’, and ‘cellular component organization or biogenesis’, were highly abundant (Figure S4). We next performed GO enrichment analysis to further investigate the over-represented GO terms of the DV-DEGs. DEGs annotated as GO terms related to ‘regulation of size’, ‘cell wall modification’, ‘hormone-related’, and ‘growth and development’ were the most significantly enriched (Figure 3, blue, green, and cyan blocks, respectively). Hormone-related GO terms and auxin-related terms such as ‘auxin mediated signaling pathway’, ‘response to auxin stimulus’, and ‘auxin transport’ were significantly enriched (Figure 3, pink block). In addition, the DEGs associated with the regulation of secondary metabolic processes such as ‘lipid metabolic’, ‘lignin metabolic processes’, and ‘flavonoid biosynthesis’ were also significant. There were also some enriched GO terms correlated with the response to light (UV and blue light) and stress (biotic and abiotic). These results imply that auxin signaling, cell wall expansion, and developmental processes may be associated with the differential growth of dorsal–ventral petal size and shape, while secondary metabolic pathways probably contribute to asymmetric pigmentation patterns in the dorsal and ventral petals.

### 2.5. Auxin Signaling Transduction Genes Identified as Major DV-DEGs

**Figure 4:**
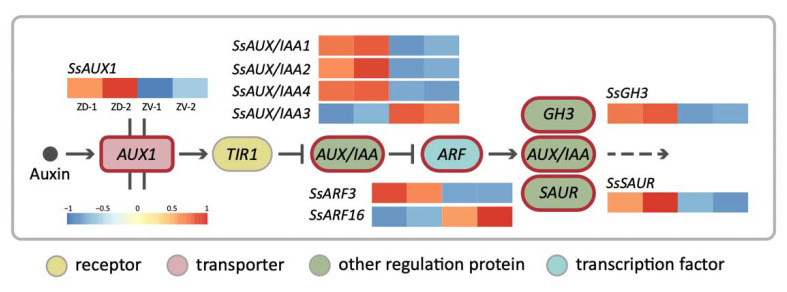
The simplified auxin signaling transduction pathway for the DV–DEGs involved in asymmetric dorsal–ventral petal growth of *S. speciosa* ‘ES’. Heatmaps represent the degree of dorsal–ventral expression differences. Colors from red to blue represent high to low expression levels.

**Figure 5:**
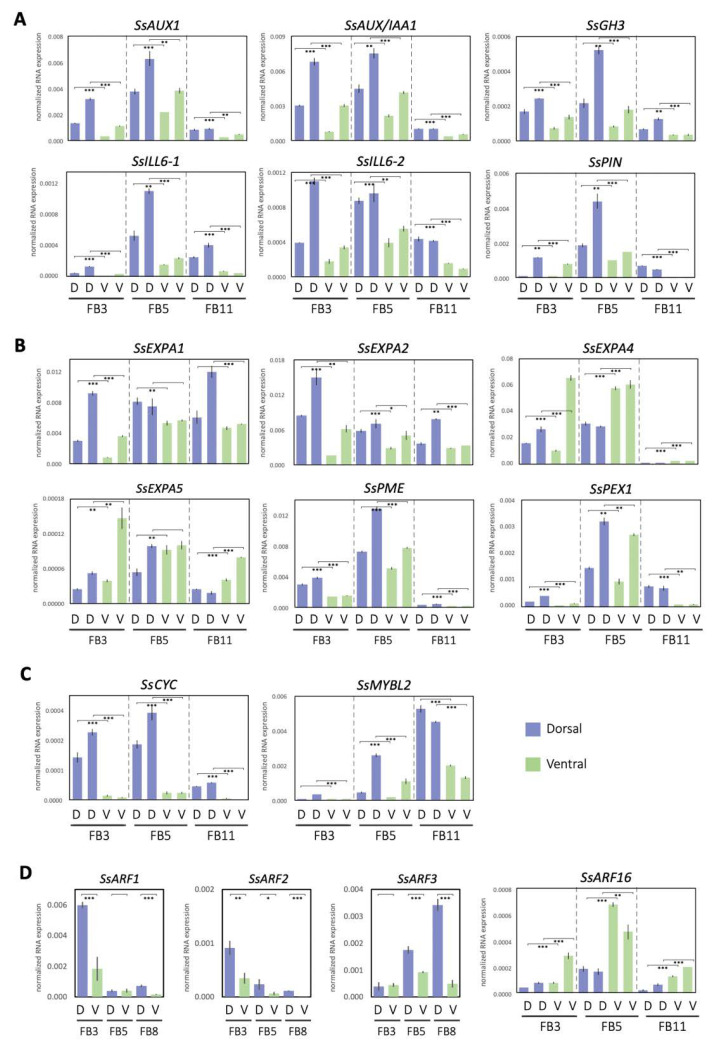
Expression patterns of asymmetric petal DV-DEGs at developmental stages FB3, FB5, and FB8/11, validated by qRT-PCR. DEGs involved in processes of annotating (**A**) the auxin pathway, (**B**) cell wall modification, (**C**) floral symmetry, and (**D**) auxin response factors. D, dorsal petals; V, ventral petals. Data in (**A**–**D**) are the mean values from two independent experiments, except for *SsARFs*, which is from three independent experiments. The mean values ± SD are from three technical repeats. Statistical analysis between the paired dorsal and ventral petal samples was determined by Student’s paired *t* test. Paired samples are connected by lines bracketed at both ends. Statistical analysis between the paired dorsal and ventral petal samples was determined by Student’s *t* test. * *p* < 0.05, ** *p* < 0.01, *** *p* < 0.001.

To further understand the biological functions of the DV-DEGs, pathway-based analysis was conducted. A total of 301 DV-DEGs (47.8%) were successfully annotated to 177 KEGG pathways. Notably, 18 DV-DEGs were assigned to hormone signaling transduction pathways (Table S4). A total of 9 of these 18 DV-DEGs could be mapped to auxin signaling transduction pathway genes, including homologs of *AUXIN1/LIKE-AUX1 (AUX1/LAX)*, *Auxin/INDOLE-3-ACETIC ACID (Aux/IAA)*, *AUXIN RESPONSE FACTOR* (*ARF*), auxin-responsive *Gretchen Hagen 3* (*GH3*), and small auxin-up RNA gene family (*SAUR*) (Figure 4). The expression patterns of these DV-DEGs were validated by qRT-PCR with a focus on FB3 (initiating zygomorphy), FB5 (zygomorphy establishing), and FB8/FB11 (zygomorphy fully developed) stages (Figure 5A,D).

The qRT-PCR results of selected DEGs were quite consistent with the gene expression profiles of RNA-Seq (Supplementary Table S8). Among the 27 DV-DEGs we validated, 26 of them shared the same DV expression patterns between RNA-Seq and qRT-PCR, supporting the accuracy of RNA-Seq. Since only two biological replicates were used in RNA-Seq, genes that did not show a large differential expression between dorsal and ventral petals may not have been identified. Most of these signaling transduction pathway DEGs including *SsAUX1*, *SsAUX/IAAs*, *SsARF2*, and *SsGH3* were found to be dorsally highly expressed, whereas only *SsARF16* had a high ventral expression, especially at stage FB5 (Figure 5A,D). Although *SsARF1* and *SsARF3* were not identified as DV-DEGs in the comparative transcriptomes of FB5, *SsARF1* and *SsARF3* also had a high dorsal transcript at the FB3 stage and FB8 stage, respectively (Figure 5D).

Based on our reconstructed phylogeny of the *ARF* family, both *SsARF1* and *SsARF2* in clade II were close to *SsARF3*, while *SsARF3* and *SsARF16* were grouped to clade IIIc (Figure S6A). MicroRNA-target tool analysis (see Supplementary Methods) further predicted that both *SsARF2* and *SsARF16* were putative miRNA targets. *SsARF16* formed a near complementary sequence pair with miR160 with a 1 nt mismatch at the 3′ end, while *SsARF3* base-paired with *TRANS-ACTING SIRNA3* (*TAS3)*-derived trans-acting short-interfering RNA (ta-siRNA) (Figure S6B). This implied that the small RNA regulatory mechanism on auxin response may also be involved in dorsal–ventral petal asymmetric growth.

Apart from auxin signaling transduction, auxin conjugation and deconjugation genes were also among the dorsally highly expressed DEGs. *SsILL6-1* and *SsILL6-2* were classified in the amidohydrolase *ILR1-like 6* (*ILL6*) group for deconjugating auxin amides, whereas *SsGH3* was in the group II *GH3* family that catalyzes the synthesis of IAA amide conjugates in the regulation of free cellular auxin levels. The homeostasis of auxin storage and release was thus also associated with dorsal–ventral petal asymmetric growth.

While many auxin pathway genes were found to be DV-DEGs, very few other hormone pathways genes were identified in DV-DEGs (Figure S7). These include *SsGID1* (GA receptor), *SsABA2* (ABA biosynthesis), and *SsOPR3* (JA biosynthesis). They also showed dorsally high transcriptions (Figure S8).

### 2.6. Cell Wall Growth Genes Identified in DV-DEGs

The cell wall modification pathway is a putative response to auxin for regulating cell growth. We also identified several major cell wall modification pathway genes among the DV-DEGs. These included *SsEXPs* (*EXPANSIN*), *SsXTH* (xyloglucan endo transglucosylase/hydrolases), *SsPEX1* (pollen-specific *LRR/EXTENSIN1*), and *SsPME* (pectin methylesterase). They encode proteins homologous to cell wall modulators such as structural proteins and proteins that modify interactions related to the regulation of auxin-induced cell wall loosening during cell growth [23]. Specifically, there were six *SsEXP* family members identified in the DV-DEGs (Table S5). The *SsEXP* genes were in clade I (*EXPA1* and *EXPA2*), clade IV (*EXPA4*, *EXPA5*, and *EXPA6*), and clade III (*EXPA3*) of the *EXPA* subfamily based on our reconstructed phylogeny (Figure S9).

qRT-PCR validation of these DV-DEGs revealed that most of these cell wall modification genes were dorsally highly expressed. It was found that two *SsEXP* family genes, *SsEXPA1* and *SsEXPA2*, and two cell wall modification genes, *SsPME* and *SsPEX1*, had a high dorsal expression pattern, while two *SsEXP* genes, *SsEXPA4* and *SsEXPA5*, were ventrally high (Figure 5B).

### 2.7. Transcription Factors and CYC-RAD Modules Identified in DV-DEGs

To identify the possible master regulators responsible for asymmetric dorsal–ventral petal growth in *S. speciose* ‘ES’, we performed transcription factor (TF) predictions using the iTAK v1.6 and the Plant Transcription Factor Database v4.0 (see Supplementary Methods) of these DV-DEGs. A total of 42 putative differentially expressed TF genes (DETFs) were predicted. This included 20 and 22 DETF genes that were expressed at high levels in the dorsal and ventral petals, respectively. As expected, homologs of floral symmetry genes *CYC* (*SsCYC*) and *RAD* (*SsRAD1* and *SsRAD2*) were all identified in the DETF, with higher expression in the dorsal petals (Table S6; Figure S2A,C and Figure 5C). Phylogenetic analysis revealed that *SsRAD2* was clustered in the *RAD2* clade, homologous to *RAD* from *A. majus*, while *SsRAD1* was in the distantly related *RAD1* clade (Figure S10A). *SsRAD2* has two TCP binding sites in the 5′ regulatory region (Figure S10B), which suggests that *SsRAD2* could be a putative downstream target of SsCYC in the *SsCYC-SsRAD* regulatory module. The other six most abundant TF families represented in the DV-DEGs were *AP2/ERF-ERF* (5 DEGs), *WRKY* (4), *MYB* (3), *TIFY* (3), *C2H2* (3), and *B3* (3) (Table S6), all of which are known to function in regulating cell growth for plant development.

### 2.8. Transcription Factors of Anthocyanin Biosynthesis Identified in DV-DEGs

We also identified DV-DEGs that encode transcription factors that regulate the flavonoid biosynthesis pathway (Figure 3). In particular, an MYB transcription factor containing the MYBL2-like domain, *SsMYBL2*, was found to be mainly expressed in dorsal petals at the FB5 and FB11 stages (Figure 5C) when the dorsal corolla turned from green to purple. *MYBL2* has been reported to be the major regulator for anthocyanin synthesis/flavonoid biosynthesis [24]. In addition, several structural genes in the flavonoid and anthocyanin biosynthetic pathways, such as *SsFLS* (flavonol synthase), *SsF3′H* (flavonoid 3′-hydroxylase), and *SsGT* (UDP-glucose: flavonoid 3-O-glucosyltransferase), were also identified in the DV-DEGs (Figure S11).

### 2.9. SsCYC Localized to Nucleus and Regulated Petal Size in Nicotiana

**Figure 6:**
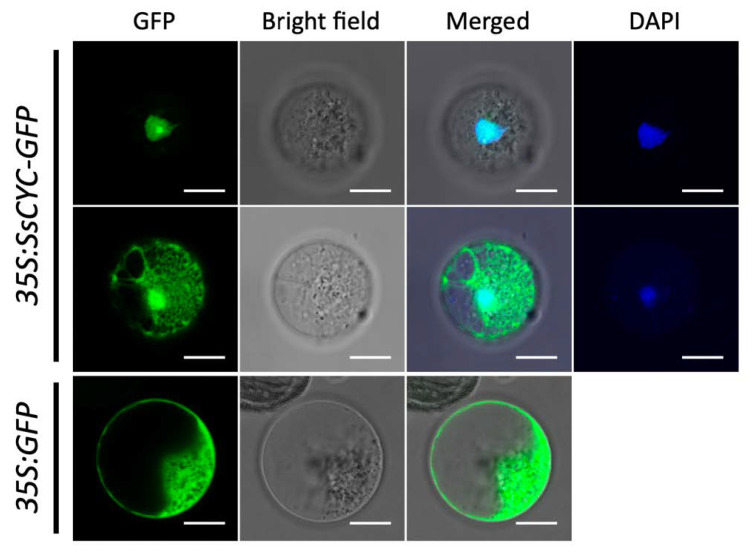
Subcellular localization of the SsCYC-GFP fusion protein in petal protoplasts of *S. speciosa* ‘ES’. The fluorescence emissions from GFP and DAPI are indicated by green and blue, respectively. Scale bars = 10 µm.

As a major regulator for floral symmetry, we asked whether the SsCYC protein acts as a TF to enter the nuclear region. Using the petal protoplast transient expression system, we found that more than 95% of the fluorescence signal was clearly localized to the nucleus in the *35S:SsCYC-GFP* transfected cells (Figure 6, upper row). Notably, 4.87% of the fusion protein was also located in the nucleus and cytoplasm (Figure 6, middle row), similar to a previous report in strawberries which found that the TCP protein can also localize to the cytoplasm [25].

**Figure 7:**
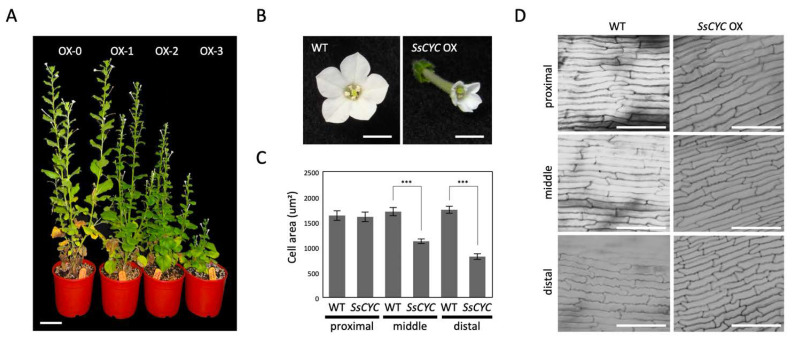
Phenotypes of *35S::SsCYC* T_1_ transgenic plants of *Nicotiana benthamiana*. (**A**) Different degrees of abnormality among the four *SsCYC* OX transgenic lines. Abnormality was ranked from OX-0 to OX-3, representing no visible differences from wildtype (0) and weak (1), medium (2), and severe phenotypes (3), respectively. Scale bar = 5 cm. (**B**) Flower morphology at the fully elongated stage. Scale bars = 0.5 cm. (**C**) The measurement of epidermal cell area in the proximal, middle, and distal parts of the corolla tube. The mean values ± SD are from three technical repeats. Each measurement has 20 cells from a single T_0_ plant. Statistical analysis was determined by Student’s *t* test, *** *p* < 0.001. (**D**) SEM of epidermal cells in the proximal, middle, and distal parts of the corolla tube at approximately the blooming stage. Scale bars = 100 um.

Given the fact that most of the DV-DEGs identified in *SsCYC* that were highly expressed in the floral bud stage are those genes relating to auxin signaling and cell growth, we hypothesized that *SsCYC* and auxin play a combined role in determining dorsal–ventral petal size and/or shape differences. To investigate *SsCYC*’s function in petal growth, we ectopically expressed it in transgenic plants of *N. benthamiana*. Genomic insertions were validated by PCR, and elevated mRNA levels of *SsCYC* were confirmed in transgenic lines (Figure S12). *35S:SsCYC* transgenic plants generally had various degrees of dwarf phenotypes (OX-0~OX-3) (Figure 7A). Noticeably, all petals of the transgenic plants were greatly reduced in size with narrower lobes towards their distal ends (Figure 7B). The measurement of petal epidermal cells in transgenic flowers revealed that cell areas were smaller (969.14 ± 49.19 µm^2^ and 721.03 ± 37.75 µm^2^) in the middle and distal regions compared to wildtype flowers (1483.13 ± 78.60 µm^2^ and 1524.65 ± 59.49 µm^2^), but not in the proximal region (Figure 7C,D). This suggests that *SsCYC* may function to repress cell expansion to affect petal size and shape.

### 2.10. Transient Over-Expression of the SsCYC in Petal Protoplasts

Since we identified DV-DEGs involved in the auxin pathway at the key bud stage when *SsCYC* was also highly expressed, we also tested whether transient over-expression of *SsCYC* can regulate these DV-DEGs in petal protoplasts. The expression levels of *SsRADs*, auxin homeostasis, auxin signaling, and cell growth genes were examined when *35S:SsCYC* was transiently over-expressed in petal protoplasts of *S. speciose* ‘ES’. Including *SsRAD2*, the expression of one auxin signaling gene (*SsAUX/IAA1*), one auxin homeostasis gene (*SsILL6-2*), and two cell growth genes (*SsEXPA1* and *SsEXPA4*) was upregulated with *SsCYC* over-expression (Figure S13A–F). However, the expression of other auxin pathway genes such as *SsILL6-1*(auxin homeostasis), *SsARF2* (auxin response factor), and one cell growth gene (*SsEXPA2*) was not induced by *SsCYC* over-expression (Figure S13G–I). Therefore, it remains inconclusive as to whether *SsCYC* regulates the auxin pathway and cell growth genes.

### 2.11. Endogenous Auxin Level Exhibited a Dorsally High Pattern

**Figure 8:**
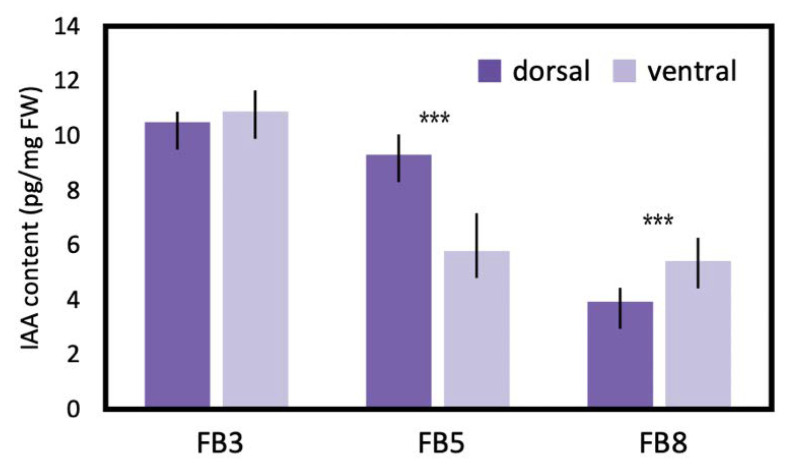
IAA levels measured by LC/MS between dorsal and ventral petals during flower bud stages FB3 (initiating zygomorphy), FB5 (zygomorphy establishing), and FB8 (zygomorphy fully developed) in *S. speciosa* ‘ES’. Each biological repeat included at least three floral buds at each stage. The mean values ± SD are from three biological repeats. Comparisons between the two groups were performed using a Student’s *t* test, *** *p* < 0.001.

To estimate the difference in endogenous auxin levels between the dorsal and ventral petals during floral zygomorphy development, IAA was extracted from FB3, FB5, and FB8 (Figure 8). IAA levels showed no difference between dorsal and ventral petals at FB3 when dorsal-ventral petal asymmetry was just about to initiate. However, at FB5 when zygomorphy was establishing (Figure 1D), the IAA level was significantly higher in dorsal petals than in ventral petals (twice as much as that in the ventral petals). This correlated with the fact that *SsCYC* also showed the highest dorsal expression at stage FB5 (Figure 5C). The difference in IAA levels between dorsal and ventral petals was no longer obvious at FB8 when flower zygomorphy was fully developed. Similarly, the DV differential expression pattern of *SsCYC* had almost disappeared by stage FB8. This indicates that the dorsal–ventral auxin level difference correlated with asymmetric dorsal–ventral petal growth, and also the DV differential expression of *SsCYC* during floral zygomorphy development.

## 3. Discussion

### 3.1. Expression of the Auxin Pathway and Cell Growth Genes Correlate to DV Petal Asymmetry

Our transcriptomic analysis identified major auxin signaling pathway genes which are dorsally highly expressed, and this was associated with a longer dorsal tube length (Figure 1D,E and Figure 4). Larger petal size has been shown to be enhanced by auxin via the triggering of auxin signaling pathway genes, both in *Arabidopsis* and orchids [26]. Our qRT-PCR validation revealed that major auxin homeostasis (two *SsILL6s* and one *SsGH3*), transport (*SsPIN* and *SsAUX1*), signaling (*SsAUX/IAAs* and *SsARF2*), and early auxin response (*SsSAUR*) genes all had high dorsal expression levels in bud stage FB3 (zygomorphy initiating), but their expression increased even further at FB5 (zygomorphy establishing) (Figure 5A). However, the difference in their expression in ventral petals became less evident towards FB11 (when zygomorphy was already fully developed). Similarly, the expression of *SsCYC* also exhibited high dorsal expression from FB3 to FB5, but this trend was no longer evident at FB11 (Figure 5C). The expression of auxin signaling pathway genes thus correlates with the expression of *SsCYC*, particularly during the zygomorphy establishing stage (FB5) (Figure 1D). These results suggest that auxin pathway regulation, together with *SsCYC*, is involved in DV petal asymmetry.

*EXP* family genes have been reported to be regulated by auxin signaling to control cell growth (cell wall modification) by promoting cell expansion [23]. Our DV-DEG and qRT-PCR validation also found that two of these cell growth genes (*SsEXP1* and *SsEXP2*) had high dorsal expression (Figure 5B). Although this suggests that the activation of auxin signaling in dorsal petals may mediate larger cell growth, this is arguable because there were also two *SsEXPs* genes (*SsEXP4* and *SsEXP5*) that exhibited high ventral expression (Figure 5B). One possible explanation could be that these *SsEXPs* genes each regulate the local outgrowth of corolla tubes such as the dorsal gibbous structure and ventral ventricose chamber. Previously, a phenotype–genotype association from geometric morphometric analysis suggested that the functional *SsCYC* allele correlates with the dorsal gibbous structure and ventral ventricose chamber [21]. These *SsEXPs* may act similarly to *SsCYC* in controlling these local outgrowths of the corolla tube. It would be a good idea in the future to check whether the expression of these *SsEXPs* is concentrated in these petal outgrowth regions.

### 3.2. Auxin Dorsal–Ventral Asymmetric Distribution Correlate to DV Petal Asymmetry

Local micro-applications of auxin to floral primordia have been shown to convert typical zygomorphic flowers to ventralized actinomorphic peloric flowers in *A. majus*, probably because of the disturbance of normal endogenous auxin distribution, resulting in transient local auxin concentrations that may vary in floral primordia [27]. In Asteraceae species *Matricaria inodora*, the spraying of auxin on the capitulum inflorescence can convert the actinomorphic disc florets into zygomorphic ray florets by enhancing the expression of the *CYC* homolog (*RAY2*) [28]. Recently, Liu et al. [29] identified one putative ARE (auxin responsive element) site in a *CpCYC* promoter of *Chirita pumila*. The EMSA result showed that this ARE site can be bound by floral nucleoproteins, implying that auxin may regulate *CpCYC* expression. These findings suggest that auxin may regulate dorsal–ventral petal asymmetry through the activation of *CYC.*

Our finding of a higher endogenous auxin level in dorsal petals thus provides new insight that the asymmetric distribution of auxin may regulate asymmetric dorsal–ventral petal growth. At bud stage FB3, when petal zygomorphy is just about to develop, there is no difference in endogenous auxin levels between dorsal and ventral petals (Figure 8). However, at bud stage FB5, the auxin in dorsal petals increased to a significantly higher level, which coincides with the key stage of establishing petal zygomorphy (Figure 1D and Figure 8). This also correlates with the highest *SsCYC* expression level at bud stage FB5 (Figure 5C). We further tested the hypothesis that *SsCYC* regulates auxin pathway genes. However, when we transiently over-expressed *SsCYC* in petal protoplasts, not all selected auxin pathway and cell growth genes were activated by *SsCYC* (Figure S13). This suggests that over-expressing *SsCYC* was not able to activate the expression of most of the auxin pathway genes. Thus, it is less likely that *SsCYC* regulates the auxin pathway. Rather, our results, together with the prediction from previous studies, suggest that auxin may regulate dorsal–ventral petal asymmetry, perhaps by promoting *SsCYC* expression.

In the future, we will treat auxin or auxin inhibitor on flower buds of *S. speciosa* ‘ES’ and subsequently examine whether *SsCYC* can be upregulated or downregulated. Moreover, we will examine whether the auxin level is increased in *SsCYC*-over-expressing *Nicotiana* flowers. These studies will help to clarify whether auxin and *SsCYC* work dependently or independently in determining zygomorphy.

### 3.3. SsCYC May Regulate Petal Size in a Context-Dependent Manner

Our results show that ectopically expressing *SsCYC* in *Nicotiana* resulted in a reduction in petal size by repressing cell expansion (smaller cell area, see Figure 7B,C). This is similar to the result from another *S. speciosa* cultivar (‘Pink Flower’, ‘PF’) that shows that the over-expression of *SsCYC* in *A. thaliana* also repressed petal cell expansion [19]. However, this is different from the observation in *S. speciosa* ‘ES’ itself, where the *SsCYC*-expressed dorsal petals have longer tube lengths due to the expanded epidermal cell size in the proximal part of the dorsal tube (Figure 1E and Figure 2C,D,K,L). This can be explained by the fact that the regulation of *CYC* has been suggested as being context-dependent [30]. In different species and tissues, *CYC* might function in protein complexes by incorporating different co-factors to activate or repress genes, thus controlling cell expansion or cell proliferation. Indeed, in *Antirrhinum majus*, the expression of *AmCYC* promotes dorsal petal growth by increasing levels of cell expansion [8], while in *Iberis amara*, *IaCYC* reduces petal cell growth by repressing cell proliferation [16]. *CYC* may also recruit different downstream targets in different lineages. For example, *CYC* has been documented to activate *RAD* for *A. majus*, but *RAD* was not found downstream of *TCP1* (*CYC* homolog) for *A. thaliana* [17,31]. Therefore, it is possible that *SsCYC* still plays a role in regulating dorsal–ventral asymmetry in *S. speciosa* ‘ES’, since a strong association between *SsCYC* expression pattern (Figure 5D) and floral asymmetry development (Figure 1E) was observed, and more importantly, some of the DV-DEGs were induced by *SsCYC* over-expression (Figure S13A–F). However, *SsCYC* might have activated different downstream targets between *S. speciosa* ‘ES’ and *Nicotiana* because of context-dependent regulation.

### 3.4. SsARF Genes May Function to Modulate Petal Size and Shape

Auxin response factors (ARFs) are TFs that transduce the auxin signal, and each ARF has its own distinct function and expression pattern in organ development. In the *Phalaenopsis* orchid, several *PeARFs* were found to be differently expressed between flowers of zygomorphic wildtype and peloric actinomorphy during the stage of floral symmetry establishment [32]. Among the DV-DEGs of the auxin pathway, we identified *SsARF2*, which showed high dorsal expression at FB5 (zygomorphy establishing) to FB8 (Figure 5D). As a higher auxin level was detected in the dorsal petals at FB5 (Figure 8), the upregulation of *SsARFs* may act in response to auxin for establishing zygomorphy.

*ARF3* has been well-characterized by its roles in organ polarity as a leaf abaxial-determining gene [33]. *TAS3* ta-siRNA-mediated regulation of *ARF3*/*ARF4* plays a critical role in establishing leaf adaxial–abaxial polarity [34]. Preliminary data shows that ta-siARF is expressed in ventral petals, and thus perhaps has a role in eliminating *ARF3* expression in the ventral side. It remains to be explored whether the development of dorsal–ventral petal asymmetry shares a similar mechanism of leaf adaxial–abaxial polarity in *S. speciosa* ‘ES’.

The role of *ARF3* in flower development has been reported to function in retarding petal growth and disrupting petal fusion into corolla [35,36]. Over-expression of tomato *ARF3* siRNA-resistant form (35S: *mSlARF3*) in tobacco, *Nicotiana benthamiana*, in *wiry* tomato (a siRNA mutant), and in *Mimulus lewisii* caused the whole flower to become smaller in size, and the petals to become narrowly unfused and separated [35,36]. Thus, *ARF3* in different angiosperm lineages has common activities in regulating petal growth.

Our results also reveal that *SsARF16* is a ventrally expressed DV-DEG (Figure 5D), which is phylogenetically grouped with *A. thaliana ARF10*, *ARF16*, and *ARF17* (Figure S6A). These three *ARF* genes have been reported to be regulated by miR160 to control petal size and shape in *Arabidopsis* [37]. When the miR160-resistant *ARF* genes are driven by their own promoters (e.g., *Pro_ARF16_*:*mARF16*), the flowers of the transgenic plants have defects with inwards-curled petals, suggesting that *ARF16* negatively regulates cell division and cell expansion.

### 3.5. SsMYBL2, a Potential Regulator of Anthocyanin Biosynthesis, May Contribute to Asymmetric Dorsal–Ventral Petal Pigmentation Pattern

The flowers of *S. speciosa* ‘ES’ display a distinct dorsal–ventral petal pigmentation pattern after zygomorphy is established from FB10 to FB16 (Figure 1D). The violet to purple corolla coloration has been reported to be mainly due to the presence of anthocyanins in *Sinningia* species [6,38]. This relates to our findings that *SsMYBL2*, a potential regulator of the anthocyanin biosynthesis pathway gene, was expressed more in dorsal petals in FB3 and FB5, but that the highest DV expression difference was at FB11 (Figure 5C). FB11 is a stage at which zygomorphy is fully developed. Thus, the development of asymmetric DV petal pigmentation is perhaps a downstream regulator of flower zygomorphy.

*SsMYBL2* is a MYB–bHLH–WD40 (MBW) transcriptional activation complex that is highly conserved across angiosperms and includes members of the R2R3-MYB or R3-MYB, bHLH, and WDR transcription factor gene families [39,40,41]. In *Arabidopsis*, R3- and R2R3-MYBs are reported to interact with AtTCP3 to form various combinations of TCP-MYB heterodimers that activate anthocyanin accumulation [42]. A recent finding in *Torenia fournieri* suggests that dorsally expressed TfCYC2 can directly regulate *TfMYB1*, which promotes anthocyanin pigmentation in the epidermal cells of petals, leading to the asymmetric dorsal–ventral petal pigmentation pattern [43]. In the future, it would be interesting to know whether a similar regulatory relationship between *SsCYC* and *SsMYBL2* is also established in *S. speciosa* ‘ES’.

**Figure 9:**
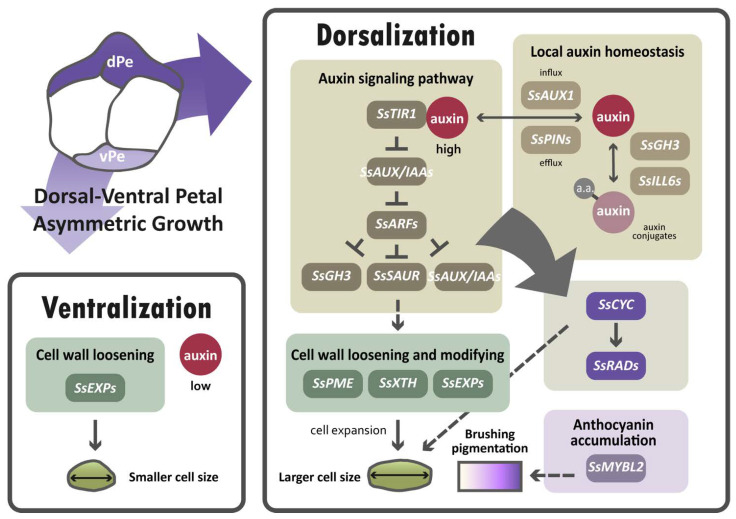
A hypothetical model proposed for the differential gene regulatory networks (GRNs) that determine dorsal–ventral petal asymmetry in zygomorphic *S. speciosa* ‘ES’. Solid lines represent direct or indirect regulation, and dashed lines indicate uncertain regulatory interactions. Dorsal petals, dPe; ventral petals, vPe.

The major finding of this paper suggests that auxin regulation is important for establishing dorsal–ventral petal asymmetry in *S. speciosa* ‘ES’, based on the results of transcriptomic analysis, the validation of DV-DEG, and the high endogenous auxin level in dorsal petals. In dorsal petals, due to the higher endogenous auxin levels detected, auxin-signaling and homeostasis genes may be upregulated in response to elevated auxin (brown box in Figure 9). This may subsequently modulate cell wall loosening genes to regulate cell size (green boxes in Figure 9). As over-expressing *SsCYC* in *Nicotiana* also affects cell expansion, *SsCYC* may act directly or be mediated by auxin to regulate petal cell size (dashed lines in Figure 9). We also found a major regulator of anthocyanin biosynthesis, *SsMYBL2,* to be highly expressed in dorsal petals, perhaps functioning to generate dorsal–ventral petal purple pigmentation differences.

## 4. Materials and Methods

### 4.1. Plant Materials

*Sinningia speciosa* ‘ES’ was originally from M. Peixoto of Mogi das Cruzes, SP, Brazil [44]. *Sinningia speciosa* ‘ES’ is a wild accession with a zygomorphic flower. A total of 120 individual plants of *S. speciosa* ‘ES’ F2 population were grown in a walk-in greenhouse at the National Taiwan University (Taipei City, Taiwan) under an LED light intensity of 200 μmol·m^–2^·s^–1^, and a 16 h light/8 h dark cycle with 70–80% relative humidity at 22–25 °C.

### 4.2. Morphological Observation

We divided floral buds into FB1 to FB16 stages according to the length of the dorsal corolla tube (Figure 1A,B). To examine petal and stamen primordia initiation, we further divided stage 1 floral buds (when the length of the dorsal tube was smaller than 2 mm) into seven sub-stages, namely FB1-1–FB1-7, using scanning electron microscopy (SEM). FB5 (when the dorsal tube length = 8–10 mm) is the key stage during which dorsal–ventral petal asymmetry is established—that is, the length and shape of the dorsal tube starts to differentiate from those of the ventral tube. FB8 is the stage at which purple color starts to accumulate in dorsal petals (tubes and lobes) but not in ventral petals. Flower buds at stages FB5 and FB8 (when the dorsal tube length = 15–18 mm) were therefore harvested to observe the epidermal cell morphology of dorsal and ventral petals by means of cryo-scanning electron microscopy (Cryo-SEM). Additional information on the SEM and Cryo-SEM methods are described in the Supplementary Materials. For tube size and cell number determination, we measured tube length following the outline curvature of the corolla tube on fresh floral samples, and counted epidermal cell numbers along the main vein of the corolla tubes after the tissue was cleared.

### 4.3. RNA Extraction, cDNA Library Construction, Sequencing, and Read Mapping

To identify GRN associated with floral symmetry, we separated RNA-Seq between the dorsal and ventral petals (comprising both the tube and lobe) at FB5 when dorsal–ventral petal asymmetry was established and *SsCYC* was highly dorsally expressed. Two dorsal petals and one ventral petal, excluding the two lateral petals of each flower, were dissected and collected. For each biological replicate, petals were sampled from 10 to 20 buds. The samples were harvested immediately and frozen in liquid nitrogen and stored at −80 °C before they were used for RNA extraction. Total RNA was extracted using Trizol Reagent according to the manufacturer’s instructions (Invitrogen, Carlsbad, CA). RNA integrity was checked using an Agilent 2100 Bioanalyzer with an RNA 6000 Chip and 2100 Expert software (Agilent Technologies). rRNA depleted mRNAs were prepared using the Ribo-Zero Gold rRNA Removal Kit. All RNA sequencing libraries were constructed using the NEBNext Ultra Directional RNA Library Prep Kit for Illumina according to the manufacturer’s instructions. Four cDNA libraries (with two biological replicates) of the dorsal (ZD-1, ZD-2) and ventral (ZV-1, ZV-2) petals were constructed for RNA-Seq. The double-strand cDNA libraries were sequenced on an Illumina Hi-Seq 2000 sequencing platform. More than 60 million paired-end reads of 150 nucleotides in length were obtained for each sample. The raw RNA-Seq data are available at the National Center for Biotechnology Information Short Reads Archive (NCBI SRA) accession number SRR16636567-SRR16636570 at the following URL: https://www.ncbi.nlm.nih.gov/Traces/study/?acc=PRJNA776263, accessed on 12 November 2021). Adaptor sequences and low-quality reads were removed from the raw reads using Trimmomatic version 0.35 [45]. Clean reads were then mapped to the predicted transcripts (31,581 predicted transcripts from 8078 assembled scaffolds) and the draft genome assembly of *S. speciosa* ‘Avenida Niemeyer’ from Rio de Janeiro city, which is closely related to *S. speciosa* ‘ES’, kindly provided by Dr. Aureliano Bombarely at Virginia Tech University (Blacksburg, VA, USA) (Bombarely and Zaitlin, unpublished data), using Bowtie2 version 2.0.6 [46] and BLAT version 34 [47], respectively. Genomic regions not annotated with the aforementioned transcripts with a maximum mapping depth greater than 10 and a length longer than 300 bps were identified as novel gene loci and were included in the following differential gene discovery (Table S9).

### 4.4. Differential Gene Expression Analysis and Functional Annotation

Transcript abundance was computed based on read counts normalized by the trimmed mean of M-values (TMM) method using the Bioconductor edgeR package [48]. Differentially expressed genes (DEGs) between dorsal and ventral petal samples (ZDs vs. ZVs) were identified if the t-test *p*-value was less than 0.05 and |log2 fold-change| > 1 (see Table S10 for the comparison table). The dorsal–ventral DEGs (DV-DEGs) were annotated on the basis of their sequence similarities with known protein annotations in public databases. Heatmaps of DEG expression were generated with the ClustVis Web tool [49]. Functional annotations were performed using Gene Ontology (GO) classification, KEGG pathways, and transcription factor prediction. The details of these experiments are described in the Supplementary Material.

### 4.5. Real-Time Quantitative PCR Validation

qRT-PCR was used to validate the relative dorsal–ventral petal expressions of newly identified DEGs from *S. speciosa* ‘ES’ (GenBank: *SsEXPA1*, MW478774; *SsAUX1*, MW478775; *SsAUX/IAA1*, MW478776; *SsGH3*, MW478777; *SsPIN*, MW478778; *SsARF16*, MW478779; *SsILL6-1*, MW478780; *SsILL6-2*, MW478781; *SsGID1*, MW478782; *SsABA2*, MW478783; *SsOPR3*, MW478784; *SsEXPA2*, MW478785; *SsEXPA4*, MW478786; *SsEXPA5*, MW478787; *SsPME*, MW478788; *SsPEX1*, MW478789; *SsMYBL2*, MW478790; *SsCYC*, MW478791; *SsTCP10*, MW478792; *SsSEP1*, MW478793; *SsAGL6*, MW478794; *SsGT*, MW478795; *Ss3’GT*, MW478796; *SsFLS1*, MW478797; *SsFLS2*, MW478798; *SsF3’H*, MW478799; and *SsARF2*, MW478800). Total RNA was extracted from the pooled dorsal and ventral petals of 2–5 floral buds collected from different individuals of *S. speciosa* ‘ES’ at stages FB3 (initiating zygomorphy), FB5 (zygomorphy establishing), and FB8/FB11 (dorsal–ventral color gradient formed) for each biological replicate (Figure 1D). RNA was treated with RQ1 RNase-Free DNase (Promega, Madison, WI, USA) to remove contaminating genomic DNA. First-strand cDNA was synthesized using MMLV reverse transcriptase (Invitrogen, Carlsbad, CA). Gene-specific primers for the DEGs (Supplementary Table S7) were designed using Primer3Plus [50]. qRT-PCR assays were performed using the CFX Connect Real-Time PCR Detection System (Bio-Rad, Hercules, CA, USA) with the KAPA SYBR^®^ FAST qPCR Master Mix (2X) Kit (Sigma-Aldrich, St. Louis, MO, USA). Two biological replicates were performed in the qRT-PCR, except for *SsARFs,* for which three biological repeats were performed. The data were analyzed using CFX Maestro Software (Bio-Rad, Hercules, CA). The expression level of *S. speciosa* 18S ribosomal RNA (*Ss18S rRNA*) was used as the internal control [51,52]. Normalized mRNA expression levels were calculated as 2^−ΔCt^.

### 4.6. S Phylogenetic Analysis of the ARF, EXP, and RAD Families

The dorsiventral DEGs annotated as encoding *ARFs, EXPs,* and *RADs* were newly identified from *S. speciosa* ‘ES’ using primers that were designed based on the sequences of cultivar of ‘AN’. The translated coding sequences were aligned by MUSCLE in MEGA5.2 with the default settings [53]. Phylogenetic relationships were reconstructed using the maximum likelihood (ML) criteria [54] (Method S1). The best-fit substitution model of each dataset was implemented in PhyML 3.0 [55]. The best-fit substitution model of each dataset was evaluated by smart model selection (SMS), which was implemented in PhyML 3.0. For inferring *ARF* relationships, 22 *AtARFs* from *A. thaliana* and 4 *SsARFs* from *S. speciosa* ‘ES’ were included, and the best-fit model was JTT + G + I + F. For the *EXPs* dataset in *S. speciosa* ‘ES’, 34 *AtEXPs* (*A. thaliana*), 11 *LeEXPs* (*Solanum lycopersicum*), 5 *NtEXPs* (*Nicotiana tabacum*), and 6 *SsEXPs* were included, and the best-fit model was the LG model. Details of *EXP* sequence information are listed in Supplementary Table S8. For the *RAD* family, 11 SsRADs and 60 *RADs* homologs from monocots and dicots were included, and the best-fit model was GTR + G + I model.

### 4.7. Subcellular Localization, Functional Characterization, and Transient Activation of SsCYC

As the *SsCYC* protein is a transcription factor, we transiently transfected a DNA construct containing *35S:SsCYC-GFP* into a petal protoplast to confirm its subcellular localization into the nuclear region. *35S:GFP* construct was also transfected as a control. The details of these procedures are described in the Supplementary Materials. To further investigate the biological function of *SsCYC*, we also created *35S:SsCYC* transgenic plants of *Nicotiana benthamiana* to characterize the effect of *SsCYC* on flower development, particularly petal growth. Confirmation of T-DNA insertion of *SsCYC* by Ti plasmid and mRNA expressions in the T_1_ transgenic plants was performed using PCR and RT-PCR. The details of the transfection procedures are provided in the Supplementary Materials (section “petal protoplast transfection system”). To explore whether certain DV-DEGs can be regulated by *SsCYC*, we transfected the *35S:SsCYC-35S:GFP* effector construct into petal protoplasts, then quantified whether the transcriptional levels of candidate genes increased or decreased (putatively induced or repressed) using qRT-PCR.

### 4.8. Endogenous IAA Determination by Liquid Chromatography Mass Spectrometry (LC/MS)

To determine the endogenous level of indole-3-acetic acid (IAA), we collected 0.1 g fresh weight each for dorsal and ventral petals. The petals were ground in liquid nitrogen then dissolved in 1 mL of cold sodium phosphate buffer (50 mM, pH 7.0) with 5 ng/mL ^13^C-IAA isotope as standard. The detailed steps followed [56]. The LC/MS (Xevo^TM^TQ-S, Waters) analysis was conducted by the Metabolomic Core Facility at the Agricultural Biotechnology Research Center, Academia Sinica, Taiwan.

## Data Availability

The data presented in this study are openly available in Supplementary Materials. The RNA-Seq data are available at the National Center for Biotechnology Information Short Reads Archive (NCBI SRA) accession number SRR16636567-SRR16636570 at the following link: https://www.ncbi.nlm.nih.gov/Traces/study/?acc=PRJNA776263, accessed on 12 November 2021.

## Supplementary Materials

The following supporting information can be downloaded at: https://www.mdpi.com/article/10.3390/ijms23042073/s1: Supplementary methods.

## References

[1] Hileman L.C. (2014). Trends in flower symmetry evolution revealed through phylogenetic and developmental genetic advances. Philos. Trans. R. Soc. B Biol. Sci..

[2] Marten-Rodriguez S., Fenster C.B., Agnarsson I., Skog L.E., Zimmer E.A. (2010). Evolutionary breakdown of pollination specialization in a Caribbean plant radiation. New Phytol..

[3] Chen K.H., Lu J.Y., Wang C.N. (2019). Effective pollination of *Aeschynanthus acuminatus* (Gesneriaceae) by generalist passerines, in sunbird-absent East Asia. Sci. Rep..

[4] Chen Y.-Y., Nishii K., Kidner C., Hackett C.A., Möller M. (2020). QTL dissection of floral traits in *Streptocarpus* (Gesneriaceae). Euphytica.

[5] Perret M., Chautems A., Spichiger R., Barraclough T.G., Savolainen V. (2007). The geographical pattern of speciation and floral diversification in the neotropics: The tribe sinningieae (gesneriaceae) as a case study. Evolution.

[6] Ogutcen E., Durand K., Wolowski M., Clavijo L., Graham C., Glauser G., Perret M. (2020). Chemical Basis of Floral Color Signals in Gesneriaceae: The Effect of Alternative Anthocyanin Pathways. Front. Plant Sci..

[7] SanMartin-Gajardo I., Sazima M. (2005). Chiropterophily in Sinningieae (Gesneriaceae): *Sinningia brasiliensis* and *Paliavana prasinata* are bat-pollinated, but *P. sericiflora* is not. Not yet?. Ann. Bot..

[8] Luo D., Carpenter R., Copsey L., Vincent C., Clark J., Coen E. (1999). Control of organ asymmetry in flowers of Antirrhinum. Cell.

[9] Luo D., Carpenter R., Vincent C., Copsey L., Coen E. (1996). Origin of floral asymmetry in Antirrhinum. Nature.

[10] Raimundo J., Sobral R., Bailey P., Azevedo H., Galego L., Almeida J., Coen E., Costa M.M.R. (2013). A subcellular tug of war involving three MYB-like proteins underlies a molecular antagonism in Antirrhinum flower asymmetry. Plant J..

[11] Corley S.B., Carpenter R., Copsey L., Coen E. (2005). Floral asymmetry involves an interplay between TCP and MYB transcription factors in Antirrhinum. Proc. Natl. Acad. Sci. USA.

[12] Hsin K.T., Lu J.Y., Moller M., Wang C.N. (2019). Gene duplication and relaxation from selective constraints of *GCYC* genes correlated with various floral symmetry patterns in Asiatic Gesneriaceae tribe Trichosporeae. PLoS ONE.

[13] Hsin K.T., Wang C.N. (2018). Expression shifts of floral symmetry genes correlate to flower actinomorphy in East Asia endemic *Conandron ramondioides* (Gesneriaceae). Bot. Stud..

[14] Hsu H.J., He C.W., Kuo W.H., Hsin K.T., Lu J.Y., Pan Z.J., Wang C.N. (2018). Genetic Analysis of Floral Symmetry Transition in African Violet Suggests the Involvement of Trans-acting Factor for CYCLOIDEA Expression Shifts. Front. Plant Sci..

[15] Cubas P., Lauter N., Doebley J., Coen E. (1999). The TCP domain: A motif found in proteins regulating plant growth and development. Plant J..

[16] Busch A., Zachgo S. (2007). Control of corolla monosymmetry in the Brassicaceae *Iberis amara*. Proc. Natl. Acad. Sci. USA.

[17] Costa M.M., Fox S., Hanna A.I., Baxter C., Coen E. (2005). Evolution of regulatory interactions controlling floral asymmetry. Development.

[18] Yang X., Pang H.B., Liu B.L., Qiu Z.J., Gao Q., Wei L., Dong Y., Wang Y.Z. (2012). Evolution of double positive autoregulatory feedback loops in CYCLOIDEA2 clade genes is associated with the origin of floral zygomorphy. Plant Cell.

[19] Dong Y., Liu J., Li P.W., Li C.Q., Lu T.F., Yang X., Wang Y.Z. (2018). Evolution of Darwin’s Peloric Gloxinia (*Sinningia speciosa*) Is Caused by a Null Mutation in a Pleiotropic TCP Gene. Mol. Biol. Evol..

[20] Hsu H.C., Chen C.Y., Lee T.K., Weng L.K., Yeh D.M., Lin T.T., Wang C.N., Kuo Y.F. (2015). Quantitative analysis of floral symmetry and tube dilation in an F-2 cross of *Sinningia speciosa*. Sci. Hortic..

[21] Hsu H.C., Wang C.N., Liang C.H., Wang C.C., Kuo Y.F. (2017). Association between Petal Form Variation and CYC2-like Genotype in a Hybrid Line of *Sinningia speciosa*. Front. Plant Sci..

[22] Wang C.N., Hsu H.C., Wang C.C., Lee T.K., Kuo Y.F. (2015). Quantifying floral shape variation in 3D using microcomputed tomography: A case study of a hybrid line between actinomorphic and zygomorphic flowers. Front. Plant Sci..

[23] Majda M., Robert S. (2018). The Role of Auxin in Cell Wall Expansion. Int. J. Mol. Sci..

[24] Chen L., Hu B., Qin Y., Hu G., Zhao J. (2019). Advance of the negative regulation of anthocyanin biosynthesis by MYB transcription factors. Plant Physiol. Biochem..

[25] Wei W., Hu Y., Cui M.Y., Han Y.T., Gao K., Feng J.Y. (2016). Identification and Transcript Analysis of the TCP Transcription Factors in the Diploid Woodland Strawberry *Fragaria vesca*. Front. Plant Sci..

[26] Chen W.H., Hsu W.H., Hsu H.F., Yang C.H. (2019). A tetraspanin gene regulating auxin response and affecting orchid perianth size and various plant developmental processes. Plant Direct.

[27] Bergbusch V.L. (1999). A note on the manipulation of flower symmetry in *Antirrhinum majus*. Ann. Bot..

[28] Zoulias N., Duttke S.H.C., Garces H., Spencer V., Kim M. (2019). The Role of Auxin in the Pattern Formation of the Asteraceae Flower Head (Capitulum). Plant Physiol..

[29] Liu J., Wu J., Yang X., Wang Y.-Z. (2021). Regulatory pathways of CYC-like genes in patterning floral zygomorphy exemplified in *Chirita pumila*. J. Syst. Evol..

[30] Preston J.C., Hileman L.C. (2009). Developmental genetics of floral symmetry evolution. Trends Plant Sci..

[31] Baxter C.E., Costa M.M., Coen E.S. (2007). Diversification and co-option of RAD-like genes in the evolution of floral asymmetry. Plant J..

[32] Huang J.Z., Lin C.P., Cheng T.C., Chang B.C., Cheng S.Y., Chen Y.W., Lee C.Y., Chin S.W., Chen F.C. (2015). A de novo floral transcriptome reveals clues into Phalaenopsis orchid flower development. PLoS ONE.

[33] Fouracre J.P., Poethig R.S. (2016). The role of small RNAs in vegetative shoot development. Curr. Opin. Plant Biol..

[34] Fahlgren N., Montgomery T.A., Howell M.D., Allen E., Dvorak S.K., Alexander A.L., Carrington J.C. (2006). Regulation of AUXIN RESPONSE FACTOR3 by TAS3 ta-siRNA affects developmental timing and patterning in Arabidopsis. Curr. Biol..

[35] Yifhar T., Pekker I., Peled D., Friedlander G., Pistunov A., Sabban M., Wachsman G., Alvarez J.P., Amsellem Z., Eshed Y. (2012). Failure of the tomato trans-acting short interfering RNA program to regulate AUXIN RESPONSE FACTOR3 and ARF4 underlies the wiry leaf syndrome. Plant Cell.

[36] Ding B., Xia R., Lin Q., Gurung V., Sagawa J.M., Stanley L.E., Strobel M., Diggle P.K., Meyers B.C., Yuan Y.W. (2020). Developmental Genetics of Corolla Tube Formation: Role of the tasiRNA-ARF Pathway and a Conceptual Model. Plant Cell.

[37] Wang J.W., Wang L.J., Mao Y.B., Cai W.J., Xue H.W., Chen X.Y. (2005). Control of root cap formation by MicroRNA-targeted auxin response factors in Arabidopsis. Plant Cell.

[38] Forsyth W.G.C., Simmonds N.W. (1954). A Survey of the Anthocyanins of Some Tropical Plants. Proc. R. Soc. Lond. Ser. B Biol. Sci..

[39] Koes R., Verweij W., Quattrocchio F. (2005). Flavonoids: A colorful model for the regulation and evolution of biochemical pathways. Trends Plant Sci..

[40] Lin R.C., Rausher M.D. (2020). R2R3-MYB genes control petal pigmentation patterning in *Clarkia gracilis* ssp. *sonomensis* (Onagraceae). New Phytol..

[41] Xu W., Dubos C., Lepiniec L. (2015). Transcriptional control of flavonoid biosynthesis by MYB-bHLH-WDR complexes. Trends Plant Sci..

[42] Li S., Zachgo S. (2013). TCP3 interacts with R2R3-MYB proteins, promotes flavonoid biosynthesis and negatively regulates the auxin response in *Arabidopsis thaliana*. Plant J..

[43] Su S., Xiao W., Guo W., Yao X., Xiao J., Ye Z., Wang N., Jiao K., Lei M., Peng Q. (2017). The CYCLOIDEA-RADIALIS module regulates petal shape and pigmentation, leading to bilateral corolla symmetry in *Torenia fournieri* (Linderniaceae). New Phytol..

[44] Zaitlin D., Pierce A.J. (2010). Nuclear DNA content in Sinningia (Gesneriaceae); intraspecific genome size variation and genome characterization in *S. speciosa*. Genome.

[45] Bolger A.M., Lohse M., Usadel B. (2014). Trimmomatic: A flexible trimmer for Illumina sequence data. Bioinformatics.

[46] Langmead B., Salzberg S.L. (2012). Fast gapped-read alignment with Bowtie 2. Nat. Methods.

[47] Kent W.J. (2002). BLAT—The BLAST-like alignment tool. Genome Res..

[48] Robinson M.D., Oshlack A. (2010). A scaling normalization method for differential expression analysis of RNA-seq data. Genome Biol..

[49] Metsalu T., Vilo J. (2015). ClustVis: A web tool for visualizing clustering of multivariate data using Principal Component Analysis and heatmap. Nucleic Acids Res..

[50] Untergasser A., Cutcutache I., Koressaar T., Ye J., Faircloth B.C., Remm M., Rozen S.G. (2012). Primer3--new capabilities and interfaces. Nucleic Acids Res..

[51] Cao A., Shao D., Cui B., Tong X., Zheng Y., Sun J., Li H. (2019). Screening the Reference Genes for Quantitative Gene Expression by RT-qPCR During SE Initial Dedifferentiation in Four Gossypium hirsutum Cultivars that Have Different SE Capability. Genes.

[52] Yu Y., Zhang G., Chen Y., Bai Q., Gao C., Zeng L., Li Z., Cheng Y., Chen J., Sun X. (2019). Selection of Reference Genes for qPCR Analyses of Gene Expression in Ramie Leaves and Roots across Eleven Abiotic/Biotic Treatments. Sci. Rep..

[53] Tamura K., Peterson D., Peterson N., Stecher G., Nei M., Kumar S. (2011). MEGA5: Molecular evolutionary genetics analysis using maximum likelihood, evolutionary distance, and maximum parsimony methods. Mol. Biol. Evol..

[54] Guindon S., Dufayard J.F., Lefort V., Anisimova M., Hordijk W., Gascuel O. (2010). New algorithms and methods to estimate maximum-likelihood phylogenies: Assessing the performance of PhyML 3.0. Syst. Biol..

[55] Lefort V., Longueville J.E., Gascuel O. (2017). SMS: Smart Model Selection in PhyML. Mol. Biol. Evol..

[56] Novák O., Hényková E., Sairanen I., Kowalczyk M., Pospíšil T., Ljung K. (2012). Tissue-specific profiling of the *Arabidopsis thaliana* auxin metabolome. Plant J..

