# Supplementary material for: Transcriptomic Analysis Suggests Auxin Regulation in Dorsal-Ventral Petal Asymmetry of Wild Progenitor Sinningia speciosa
Source: Int J Mol Sci. 2022 Feb 13;23(4):2073. doi: 10.3390/ijms23042073 (PMC8876764; doi:10.3390/ijms23042073)
Supplement: Supplementary file 1 [file ijms-23-02073-s001.zip › ijms-1521599-supplementary.pdf]

Supplementary Methods

Scanning electron microscopy (SEM)

Floral buds were pre-dissected to remove sepals under stereo microscope S8 APO (Leica) and then fixed in FAA solution. Tissue was dehydrated through an ethanol series (70%, 85%, 95%, and 100% twice) with each step for at least 30 min followed by an ascending acetone gradient and finally dried in a critical point dryer (Hitachi E101). Dried samples were sputter coated by using gold–palladium using Hitachi E1011I sputter. Specimens were imaged immediately using Scanning Electron Microscope Inspect S50 (FEI, Brno, Czech Republic).

Cryo-scanning electron microscopy (Cryo-SEM)

Sample preparation for Cryo-SEM was as described (Kumar et al., 2013). Fresh floral buds were dissected and loaded on a stub. Samples were frozen with liquid nitrogen slush then transferred to a sample preparation chamber at -160℃. After 5 min, the temperature was raised to -85℃ and sublimed for 15 min. After being coated with platinum at -130℃, samples were transferred to a cryo stage in an SEM chamber and observed at < -160℃ by use of Cryo-SEM (FEI Quanta 200 SEM/Quorum Cryo System PP2000TR FEI, FEI Co., Hillsboro, OR) at 20 KV.

Functional annotations of DV-DEGs

Annotation of DEGs was perform by using the blastx-fast function against the NCBI nr database of vascular plants in the Blast2GO version 5 (Conesa et al., 2005). Functional annotation and Gene Ontology (GO) classification was performed using Blast2GO and WEGO (Ye et al., 2006). A statistical analysis of GO term enrichment (false discovery rate (FDR)≤0.05) was performed using the BiNGO tool of Cytoscape_v2.8.3 (Maere et al., 2005) to infer isofunctionality from DEG homology. For ortholog assignment and pathway mapping, the KAAS (KEGG Automatic Annotation Server) was used to obtain KO (KEGG Orthology) assignments and mapped KEGG pathways (Moriya et al., 2007). Transcription factors were predicted using the iTAK v1.6 (Zheng et al., 2016) program and the Plant Transcription Factor Database v4.0 (Jin et al., 2017) via BLAST searches. DEGs belonging to plant hormone pathways and TF families were identified by manual adjustment as well according to the gene annotation.

Prediction of microRNAs and protein structure analysis

To identify microRNAs targeting *SsARF* genes, psRNATarget online database 2017 was used (the parameter was set as default) (Dai et al., 2018). All identified

microRNA sequences of *A. thaliana* and *Solanum lycopersicum* (miRBase Release 21, June 2014) were used to search for *SsARF* genes. The protein sequences of *SsARFs* and *AtARFs* were aligned and definition of domain structure were according to previous publications (Ulmasov et al., 1999; Guilfoyle and Hagen, 2001; Guilfoyle, 2015; Choi et al., 2018).

Petal protoplast transfection system

To investigate the subcellular localization of SsCYC TCP transcription factor, a CaMV *35S:SsCYC-GFP* construct was transiently transfected into protoplasts isolated from petals of *S. speciosa* 'ES' at FB10-FB12. We use fluorescent fusion protein tagging to define the subcellular distribution of SsCYC proteins. The coding sequence of *SsCYC* was cloned into the XbaI and XhoI sites in the *pUC19* vector for the GFP-tagged fusion protein construct. The plasmid DNA of *pUC19* containing *35S:SsCYC-35S:GFP* was used for transient activation of *SsCYC*. The plasmid of *pUC19-35S:GFP* only was used as negative control for both experiments. The plasmid DNA were transiently transfected into petal protoplasts by modifying protocols of PEG-Ca^{2+}-mediated transformation method (Yoo et al., 2007). Fresh cut petal strips were digested by enzyme solution (containing 1.5% Cellulase and 0.4% macerozyme) for 6 hr. 10 μg and 20 μg plasmid DNA were transfected to 2 x 10^4 protoplasts and 6.65 x 10^6 protoplasts for subcellular localization and transient activation experiments, respectively. Green fluorescence signals were detected by confocal laser scanning microscopy 16-18 hours after transformation by using Zeiss LSM 780 inverted confocal microscope (Zeiss, Japan). Incubated protoplast cells were harvested by centrifuging and frozen in liquid nitrogen, and stored at -80°C before they were used for RNA extraction.

Ectopic expression of *SsCYC* in *Nicotiana benthamiana*

The coding sequence of *SsCYC* from *S. speciosa* 'Corangola' was identified and cloned in pK2GW7.0 to produce a CaMV *35S:SsCYC* OX construct. Constructed *SsCYC* OX vector was then introduced into the *Agrobacterium tumefaciens* strain EHA105 using electroporation transfection, which was then transformed into *Nicotiana benthamiana* through *Agrobacterium tumefaciens* mediated leaf disk transformation. The *SsCYC* OX transgenic plants were screened by growth medium with kanamycin. Twelve independent lines of T_0 transgenic plants were obtained and validated by checking the insertion of *SsCYC* and *NPTII* genes (Supplementary Figure 12A). *SsCYC* gene expression of T_0 transgenic plants were analyzed by RT-PCR (Supplementary Figure 12B). In order to further examine whether cell proliferation and/or cell

expansion attributed to changes of flower size, clearing method was performed for observation of petal epidermis. Cell area of the proximal, middle and distal sites of floral tube were measured. Each measurement contains 20 epidermal cells from a single plant. The differences of cell area between WT and *SsCYC* OX transgenic plants were determined by using Student's *t*-test.

References

Choi, H.S., Seo, M., and Cho, H.T. (2018). Two TPL-Binding Motifs of ARF2 Are Involved in Repression of Auxin Responses. *Front Plant Sci* 9**,** 372. doi: 10.3389/fpls.2018.00372.

Conesa, A., Gotz, S., Garcia-Gomez, J.M., Terol, J., Talon, M., and Robles, M. (2005). Blast2GO: a universal tool for annotation, visualization and analysis in functional genomics research. *Bioinformatics* 21(18)**,** 3674-3676. doi: 10.1093/bioinformatics/bti610.

Dai, X., Zhuang, Z., and Zhao, P.X. (2018). psRNATarget: a plant small RNA target analysis server (2017 release). *Nucleic Acids Res* 46(W1)**,** W49-W54. doi: 10.1093/nar/gky316.

Guilfoyle, T.J. (2015). The PB1 domain in auxin response factor and Aux/IAA proteins: a versatile protein interaction module in the auxin response. *Plant Cell* 27(1)**,** 33-43. doi: 10.1105/tpc.114.132753.

Guilfoyle, T.J., and Hagen, G. (2001). Auxin response factors. *Journal of Plant Growth Regulation* 20(3)**,** 281-291. doi: DOI 10.1007/s003440010026.

Jin, J., Tian, F., Yang, D.C., Meng, Y.Q., Kong, L., Luo, J., et al. (2017). PlantTFDB 4.0: toward a central hub for transcription factors and regulatory interactions in plants. *Nucleic Acids Res* 45(D1)**,** D1040-D1045. doi: 10.1093/nar/gkw982.

Kumar, M.N., Jane, W.N., and Verslues, P.E. (2013). Role of the putative osmosensor Arabidopsis histidine kinase1 in dehydration avoidance and low-water-potential response. *Plant Physiol* 161(2)**,** 942-953. doi: 10.1104/pp.112.209791.

Maere, S., Heymans, K., and Kuiper, M. (2005). BiNGO: a Cytoscape plugin to assess overrepresentation of gene ontology categories in biological networks. *Bioinformatics* 21(16)**,** 3448-3449. doi: 10.1093/bioinformatics/bti551.

Majda, M., and Robert, S. (2018). The Role of Auxin in Cell Wall Expansion. *Int J Mol Sci* 19(4). doi: 10.3390/ijms19040951.

Moriya, Y., Itoh, M., Okuda, S., Yoshizawa, A.C., and Kanehisa, M. (2007). KAAS: an automatic genome annotation and pathway reconstruction

server. *Nucleic Acids Res* 35(Web Server issue)**,** W182-185. doi: 10.1093/nar/gkm321.

Ulmasov, T., Hagen, G., and Guilfoyle, T.J. (1999). Activation and repression of transcription by auxin-response factors. *Proc Natl Acad Sci U S A* 96(10)**,** 5844-5849. doi: 10.1073/pnas.96.10.5844.

Ye, J., Fang, L., Zheng, H., Zhang, Y., Chen, J., Zhang, Z., et al. (2006). WEGO: a web tool for plotting GO annotations. *Nucleic Acids Res* 34(Web Server issue)**,** W293-297. doi: 10.1093/nar/gkl031.

Yoo, S.D., Cho, Y.H., and Sheen, J. (2007). Arabidopsis mesophyll protoplasts: a versatile cell system for transient gene expression analysis. *Nat Protoc* 2(7)**,** 1565-1572. doi: 10.1038/nprot.2007.199.

Zheng, Y., Jiao, C., Sun, H., Rosli, H.G., Pombo, M.A., Zhang, P., et al. (2016). iTAK: A Program for Genome-wide Prediction and Classification of Plant Transcription Factors, Transcriptional Regulators, and Protein Kinases. *Mol Plant* 9(12)**,** 1667-1670. doi: 10.1016/j.molp.2016.09.014.

♦ Supplementary tables and figures

Supplementary Table S1. Developmental feature definition for dorsiventrally asymmetric growth in the corolla of *S. speciosa* ‘Espírito Santo’ flowers at sub-stages of FB1.

**Sub-stages**	**Developmental features**
**FB1-1**	Formation of floral meristem
**FB1-2**	Sepal primordia development
**FB1-3**	Petal primordia development (lobe)
**FB1-4**	Stamen primordia development
**FB1-5**	Carpel primordia development, petals covering stamen
**FB1-6**	Calyx tube initiation and lobe elongation
**FB1-7**	Lobe enclosed, dorsiventral lobe aestivation*
**FB1-8**	Dorsal staminode arrest growth*

*Asymmetric growth between the dorsal and ventral floral organs.

Supplementary Table S2. RNA-seq reads and mapping statistics.

**Samples**	**Raw reads**	**Clean reads**	**Mapping reads/percentage (%)**	
**ZD-1**	60,340,442	59,861,198	50,768,163	84.8
**ZD-2**	68,210,128	67,729,837	57,243,853	84.5
**ZV-1**	63,538,566	62,920,327	53,655,972	85.3
**ZV-2**	72,690,968	72,088,207	61,367,923	85.1
**Total**	264,780,104	262,599,569	223,035,911	

Supplementary Table S3. Summarized statistics of functional annotation for 630 DV-DEGs against the public databases.

Databases	No. of DEGs	Percentage (%)
**Annotated in NR**	574	91.1
**Annotated in GO**	444	70.5
**Annotated in KEGG**	224	47.8
**Total DEGs**	630	100.0

Supplementary Table S4. Summary of DEGs assigned to plant hormone signal transduction pathways.

**Gene name ('ES')**[b]	**DEG ID ('AN')**	**Expression patterns**[a]	**Pathways**	**KO numbers**	**KO genes**
SsPP2C	Sispe038Scf0983g03003	D	Abscisic acid	K14497	PP2C
SsABF	Sispe038Scf0044g00001	V	Abscisic acid	K14432	ABF
SsARF2	Sispe038Scf6188g00023	D	Auxin	K14486	ARF
SsAUX/IAA1	Sispe038Scf0308g01066	D	Auxin	K14484	AUX/IAA
SsAUX/IAA2	Sispe038Scf0403g09007	D	Auxin	K14484	AUX/IAA
SsAUX/IAA4	Sispe038Scf4222g01017	D	Auxin	K14484	AUX/IAA
SsAUX1	Sispe038Scf1393g05050	D	Auxin	K13946	AUX1
SsGH3	Sispe038Scf1875g02004	D	Auxin	K14487	GH3
SsSAUR	Sispe038Scf1202g14033	D	Auxin	K14488	SAUR
SsARF16	Sispe038Scf0608g04048	V	Auxin	K14486	ARF
SsAUX/IAA3	Sispe038Scf2893g00040	V	Auxin	K14484	AUX/IAA
SsTCH4	Sispe038Scf1008g10041	D	Brassinosteroid	K14504	TCH4
SsBRI1	Sispe038Scf1746g11005	V	Brassinosteroid	K13415	BRI1
SsEIN3	Sispe038Scf0439g00009	V	Ethylene	K14514	EIN3
SsGID1	Sispe038Scf4439g00032	D	Gibberellin	K14493	GID1
SsJAZ1	Sispe038Scf0116g02021	D	Jasmonic acid	K13464	JAZ
SsJAZ2	Sispe038Scf0228g08007	D	Jasmonic acid	K13464	JAZ
SsJAZ	Sispe038Scf6299g00006	D	Jasmonic acid	K13464	JAZ

[a]Expression patterns of DEGs with Dorsal- or Ventral-high transcript levels.

[b]Genes newly identified from *S. speciosa* 'ES' based on the draft genome sequences of cultivar of 'AN' were showed in bold.

Supplementary Table S5. Summary of DEGs annotated as members of the *EXPANSIN* family.

**DEG ID (‘AN’)**	**Gene name (‘ES’)[c]**	**Expression patterns[a]**	**Subfamily, clades**	**Annotations**
Sispe038Scf2587g00004[b]<br>Sispe038Scf0517g00048	***SsEXPA1***	D	EXPA, I	alpha-expansin 6
Sispe038Scf1947g01015	***SsEXPA2***	D	EXPA, I	expansin-A10
Sispe038Scf1393g01034	***SsEXPA3***	V	EXPA, III	expansin-A8-like
Sispe038Scf0399g01001	***SsEXPA5***	V	EXPA, IV	expansin-A4-like
Sispe038Scf0327g00028	***SsEXPA4***	V	EXPA, IV	expansin-A6
Sispe038Scf0224g00031	*SsEXPA6*	D	EXPA, IV	expansin-A6-like

[a]Expression patterns of DEGs with Dorsal- or Ventral-high transcript levels.

[b]Sispe038Scf0517g00048 and Sispe038Scf2587g00004 can be assembled into a single contig.

[c]Genes newly identified from *S. speciosa* ‘ES’ based on the draft genome sequences of cultivar of ‘AN’ were showed in bold.

Supplementary Table S6. Genes encoding 42 transcription factors predicted from the 630 dorsiventral differentially expressed genes.

**DEG ID (‘AN’)**	**Gene name (‘ES’)[b]**	**Expression patterns[a]**	**Families**	**NR annotations**
Sispe038Scf5680g00016	***SsERF1***	D	AP2/ERF	ethylene-responsive transcription factor 2
Sispe038Scf0228g08027	***SsERF17***	D	AP2/ERF	ethylene-responsive transcription factor ERF016-like
Sispe038Scf1061g02075	***SsERF3***	D	AP2/ERF	ethylene-responsive transcription factor 4-like
Sispe038Scf1948g00046		D	AP2/ERF	ethylene-responsive transcription factor ABR1-like
Sispe038Scf1783g02026		D	AP2/ERF	ethylene-responsive transcription factor 1A-like
Sispe038Scf2996g00029	***SsNGAL1***	D	B3	B3 domain-containing protein At2g36080-like
Sispe038Scf1202g13005		D	B3	B3 domain-containing protein At2g36080-like
Sispe038Scf0163g00025		V	B3	B3 domain-containing protein Os07g0679700
Sispe038Scf6188g00023	***SsARF2***	D	B3-ARF	auxin response factor 2-like
Sispe038Scf6188g00023		D	B3-ARF	auxin response factor 2-like
Sispe038Scf0608g04048	***SsARF16***	V	B3-ARF	auxin response factor 16-like
Sispe038Scf2159g01072	***SsCIB2***	D	bHLH	transcription factor bHLH62
Sispe038Scf3458g00018		V	bHLH	transcription factor bHLH62
Sispe038Scf0044g00001	*SsABF2*	V	bZIP	bZIP transcription factor TRAB1-like
Sispe038Scf0247g02018	*SsBBX15*	V	C2C2-CO-like	zinc finger protein CONSTANS-LIKE 16
Sispe038Scf5557g00020		V	C2H2	protein SHOOT GRAVITROPISM 5-like
Sispe038Scf1614g01067		V	C2H2	zinc finger protein 91-like
Sispe038Scf0893g03002		V	C2H2	hypothetical protein CDL12_14393
Sispe038Scf0439g00009		V	EIL	ETHYLENE INSENSITIVE 3-like 1 protein
Sispe038Scf1947g02019	*SsHB13*	V	HB-HD-ZIP	homeobox-leucine zipper protein ATHB-13
Sispe038Scf0266g00013	*SsHB13*	V	HB-HD-ZIP	homeobox-leucine zipper protein ATHB-13-like
Sispe038Scf2515g00020	*SsWOX1*	V	HB-WOX	WUSCHEL-related homeobox 1
Sispe038Scf2368g00005[b]	***SsAGL6***	V	MADS	MADS-box transcription factor 6
Sispe038Scf0056g05037	***SsSEP1***	V	MADS-MIKC	SEPALLATA 1-like isoform X2
Sispe038Scf1077g00028		V	MADS-MIKC	MADS-box transcription factor 6-like
Sispe038Scf1614g02066		V	MADS-MIKC	truncated transcription factor CAULIFLOWER A-like
Sispe038Scf1651g00049	***SsMYB14***	D	MYB	MYB-related transcription factor
Sispe038Scf3835g03022	***SsMYBL2***	D	MYB	transcription repressor MYB6-like
Sispe038Scf0018g22022	***SsRAD2***	D	MYB	transcription factor radialis-like
Sispe038Scf0367g01001	*SsMYBS1*	V	MYB	transcription factor MYBS1
Sispe038Scf0170g01016	***SsRAD1***	D	MYB-related	protein RADIALIS-like 3
Sispe038Scf0757g01046	*SsRVE1*	V	MYB-related	protein REVEILLE 1-like isoform X1
Sispe038Scf1202g12026	***SsOFP6***	D	OFP	transcription repressor OFP6-like
Sispe038Scf1400g01001	***SsCYC***	D	TCP	TCP transcription factor CYC1D
Sispe038Scf0165g00067	***SsTCP10***	V	TCP	transcription factor tcp14-like
Sispe038Scf0116g02021	***SsJAZ1***	D	Tify	protein TIFY 10A-like
Sispe038Scf0228g08007	***SsJAZ2***	D	Tify	protein TIFY 10A-like
Sispe038Scf6299g00006	***SsJAZ3***	D	Tify	protein TIFY 9 isoform X2
Sispe038Scf1393g02049		D	WRKY	probable WRKY transcription factor 33
Sispe038Scf2358g01033	*SsWRKY14*	V	WRKY	probable WRKY transcription factor 14
Sispe038Scf0146g00043	*SsWRKY35*	V	WRKY	probable WRKY transcription factor 14
Sispe038Scf3275g05006		V	WRKY	probable WRKY transcription factor 13

[a]Expression patterns of DEGs with Dorsal- or Ventral-high transcript levels.

[b]Genes newly identified from *S. speciosa* ‘ES’ based on the draft genome sequences of cultivar of ‘AN’ were showed in bold.

Supplementary Table S7. Selected DV-DEGs for qRT-PCR validation. GeneBank ID, Mapped scaffold, RNA-seq pattern, qRT-PCR expression pattern and list of oligonucleotide primers indicated.

Gene name	GenBank ID/Mapped scaffold	RNA-Seq pattern[a]	qRT-PCR pattern[b]	Primer sequence (5'-3')
SsAUX1	MW47884<br>Sispe038Scf1393g05050	D (suppl Fig. 5A)	D (Fig. 5A)	F: CAGTGTCACCATCCCATTCTTG<br>R: TTGATATTGATACCTCTCCCCATG
SsAUX/IAA1	MW478776<br>Sispe038Scf0308g01066	D (suppl Fig. 5A)	D (Fig. 5A)	F: GGCACATGTATGTATAATCCATGAC<br>R: GCCTCTAATACAAGCAGGAAGC
SsGH3	MW478777<br>Sispe038Scf1875g02004	D (suppl Fig. 5A)	D (Fig. 5A)	F: GGGTTGTGTCCACCTATTTCAG<br>R: TTTTCTTCTTGGGGGTGGTTGG
SsPIN	MW478778<br>Sispe038Scf0394g01023	D (suppl Fig. 5A)	D (Fig. 5A)	F: CATTTTGTTGGGCTTGTAAAGGTG<br>R: CTACATTGCGTGACTTGTAGCG
SsARF16	MW478779<br>Sispe038Scf0608g04048	V (suppl Fig. 5A)	V (Fig. 5D)	F: GGCTTTGAAACCAGACAATGC<br>R: GAGTATCGTTAGCCTTCAGGTG
SsARF2	MW478800<br>Sispe038Scf6188g00023	D (suppl Fig. 5A)	D (Fig. 5D)	F: CTCGGCCATAACTTGTGATCTC<br>R: CTCAGCTTCAATGATGCCATCC
SsILL6-1	MW478780<br>Sispe038Scf0144g08030	D (suppl Fig. 5A)	D (Fig. 5A)	F: GCACTGGGCTCGTCTATATTTTAG<br>R: CGCTTGGACATATGTTTTACTCC
SsILL6-2	MW478781<br>Sispe038Scf1052g01029	D (suppl Fig. 5A)	D (Fig. 5A)	F: CAATAGGAGCTGCTACTCATGC<br>R: CAGATCTCCATTTGCGAGCTAC
SsGID1	MW478782<br>Sispe038Scf4439g00032	D (suppl Fig. 5A)	D (suppl Fig. S8A)	F: TTCCTCCCTTCTTCTAGCAGTG<br>R: GGACGAGAGCAGCAATCAAAAG
SsABA2	MW478783<br>Sispe038Scf1007g02033	D (suppl Fig. 5A)	E (suppl Fig. S8A)	F: GGTGGTTTCACATCTGCAAACC<br>R: GCAAAACCAAACATGTAGCTCAG
SsOPR3	MW478784<br>Sispe038Scf0228g05012	D (suppl Fig. 5A)	D (suppl Fig. S8A)	F: CGCCTTTAAACATGTGTACTGC<br>R: CTTCCCACTCTCAGTATTGGAC
SsEXPA1	MW478774<br>Sispe038Scf2587g00004	D (suppl Fig. 5B)	D (Fig. 5B)	F: CCGGATTGCAAAAGTGATCTGG<br>R: AACCCTAGAGGCCCATTTGC
SsEXPA2	MW478785<br>Sispe038Scf1947g01015	D (suppl Fig. 5B)	D (Fig. 5B)	F: ACAAGTTCGAGTCGAAGTGAG<br>R: GCCCCCTTCGTTTTGATACTAG
SsEXPA4	MW478786<br>Sispe038Scf0327g00028	V (suppl Fig. 5B)	V (Fig. 5B)	F: GGTTAGCGTGAAGGGATCAAAG<br>R: GAAGGGACGATGTTCAATGAGG
SsEXPA5	MW478787<br>Sispe038Scf0399g01001	V (suppl Fig. 5B)	V (Fig. 5B)	F: AGCATAAAGGGGTCTAGGAGTG<br>R: CCAATGTGAGGGAACAATGTCC
SsPME	MW478788<br>Sispe038Scf3457g00008	D (suppl Fig. 5B)	D (Fig. 5B)	F: CCGACCCGAATCAGAATAC<br>R: GCCTAGATAAGTTGGGAAAGAA
SsPEX1	MW478789<br>Sispe038Scf3686g00006	D (suppl Fig. 5B)	D (Fig. 5B)	F: TGTGATGGTTATTATGGGTGTG<br>R: CATTATTAAGCAAGCTGAAGGAT
SsMYBL2	MW478790<br>Sispe038Scf3835g03022	D (suppl Fig. 5C)	D (Fig. 5C)	F: CATTGCCTTCCGTCCTC<br>R: AAAGTGACCAACCATAACCG
SsCYC	MW478791<br>Sispe038Scf1400g01001	D (suppl Fig. 5C)	D (Fig. 5C)	F: ACCTCACAATCCAACCTGTGTGAC<br>R: CCACAGAAACCACGCAGAATTAC
SsTCP10	MW478792<br>Sispe038Scf0165g00067	V (suppl Fig. 5C)	V (suppl Fig. S8B)	F: AGGTACAGGCTGTGATGACC<br>R: AACACTCACACACCACACAC
SsSEP1	MW478793<br>Sispe038Scf0056g05037	V (suppl Fig. 5C)	V (suppl Fig. S8B)	F: GTCGGAGGAGGGGAACA<br>R: TCTGGCTTGAACTCACAGGAT
SsAGL6	MW478794<br>Sispe038Scf2368g00005	V (suppl Fig. 5C)	V (suppl Fig. S8B)	F: AAGCATGGGTGTTGAAAGTAA<br>R: TCCGAATATCCAAAACACAAC
SsGT	MW478795<br>Sispe038Scf0169g01023	D (suppl Fig. 5C)	D (suppl Fig. 11)	F: TACGAGCGAGTTCGGTAAAG<br>R: GGCACGCCATTGGAAATAC
Ss3'GT	MW478796<br>Sispe038Scf1112g00131	D (suppl Fig. 5C)	D (suppl Fig. 11)	F: GGTGGAGAATAAAGAACCTG<br>R: CTAGTAACCAAAGCAAAGACAT
SsFLS1	MW478797<br>Sispe038Scf0448g01007	D (suppl Fig. 5C)	D (suppl Fig. 11)	F: GCCGAAGATCGTGTTTC<br>R: CCCTTGGGACCTTTAGTC
SsFLS2	MW478798<br>Sispe038Scf7957g00088	V (suppl Fig. 5C)	V (suppl Fig. 11)	F: TGTCTGCCCTTACCATTCTG<br>R: TTTGCCGTTGCTTAGTATCTC
SsF3'H	MW478799<br>Sispe038Scf0019g06010	V (suppl Fig. 5C)	V (suppl Fig. 11)	F: AACTCTTGACCGCATCTTTG<br>R: GCACACTATTACACTTGGGCTT
SsRAD2	Sispe038Scf0018g22022	-	D (suppl Fig. S2C)	F: GGTGCCATTTCCTAACTACAGG<br>R: GAAGTAGTTGGCCCATAAACCC
Ss18S		Not applicable	Internal control	F: GCGGATGTTGCTTATAGGACTC<br>R: TCAGCCTTGCGACCATACTC

[a]Expression patterns with <u>D</u>orsal- or <u>V</u>entral-high transcripts as RNA-Seq data.

[b]Expression patterns with <u>D</u>orsal-, <u>V</u>entral-high or <u>E</u>qual transcripts as qRT-PCR confirmation.

Supplementary Table S8. Homologous sequences of *EXP* genes used to reconstruct the ML tree.

Species	Sequence name	Locus	Accession number
Arabidopsis thaliana	*EXPA1*	AT1G69530	NP_849869
	EXPA2	AT5G05290	AAL36391
	EXPA3	AT2G37640	NP_181300
	EXPA4	AT2G39700	NP_181500
	EXPA5	AT3G29030	NP_189545
	EXPA6	AT2G28950	NP_180461
	EXPA7	AT1G12560	NP_172717
	EXPA8	AT2G40610	NP_181593
	EXPA9	AT5G02260	NP_195846
	EXPA10	AT1G26770	NP_001077599
	EXPA11	AT1G20190	NP_173446
	EXPA12	AT3G15370	NP_188156
	EXPA13	AT3G03220	NP_566197
	EXPA14	AT5G56320	NP_200443
	EXPA15	AT2G03090	NP_178409
	EXPA16	AT3G55500	NP_191109
	EXPA17	AT4G01630	NP_192072
	EXPA18	AT1G62980	NP_176486
	EXPA20	AT4G38210	NP_195534
	EXPA21	AT5G39260	NP_198742
	EXPA22	AT5G39270	NP_198743
	EXPA23	AT5G39280	NP_198744
	EXPA25	AT5G39300	NP_198746
	EXPA26	AT5G39290	NP_198745
	EXPB1	AT2G20750	NP_179668
	EXPB2	AT1G65680	NP_564860
	EXPB3	AT4G28250	NP_567803
	EXPB4	AT2G45110	NP_182036
	EXPB5	AT3G60570	NP_001319806
	EXPB6	AT1G65681	NP_001117554
	EXLA1	AT3G45970	NP_190183
	EXLA2	AT4G38400	NP_195553
	EXLA3	AT3G45960	NP_190182
	EXLB1	AT4G17030	NP_193436
Solanum lycopersicum	*LeEXP1*	Solyc06g051800.3.1	AAC63088
	LeEXP2	Solyc06g049050.3.1	AAC64201
	LeEXP3	Solyc03g031830.2.1	AAD13631
	LeEXP4	Solyc09g010860.3.1	AAD13632
	LeEXP5	Solyc02g088100.3.1	AAD13633
	LeEXP6		AAD13634
	LeEXP7		AAD13635
	LeEXP8	Solyc12g089380.2.1	AAG32920
	LeEXP9		CAB46492
	LeEXP10	Solyc03g115890.3.1	AAG32921
	LeEXP18		CAA06271
Nicotiana tabacum	*NtEXP1*		AAC96077
	NtEXP2		AAC96078
	NtEXP3		AAC96079
	NtEXP4		AAC96080
	NtEXP5		AAC96081
***Sinningia speciosa* 'Espirito Santo'**	*SsEXPA1*	Sispe038Scf2587g00004	
	SsEXPA2	Sispe038Scf1947g01015	
	SsEXPA3	Sispe038Scf1393g01034	
	SsEXPA4	Sispe038Scf0327g00028	
	SsEXPA5	Sispe038Scf0399g01001	

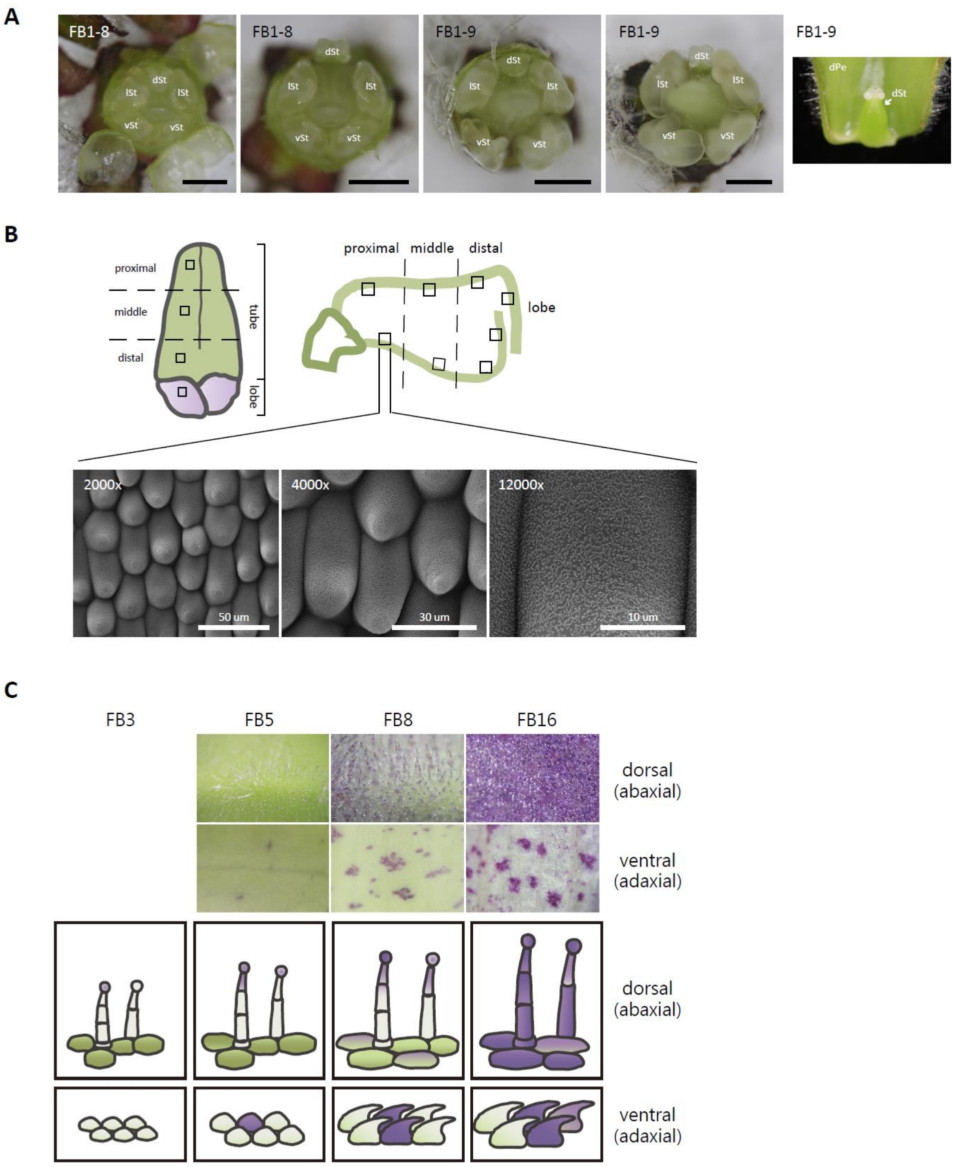

Supplementary Figure S1. Dorsiventral asymmetric features of flowers of *S. speciosa* 'Espírito Santo'. **(A)** Development of the aborted dorsal staminode at FB1-8~FB1-9 and FB3. The white arrow indicates the arrested growth of the dorsal staminode and the yellow arrowhead show the lateral nectaries.Bars = 500 µm. **(B)** Cryo-SEM of epicuticular wax on adaxial side of petal epidermis. The upper panel illustrated the sampled regions of ventral petal at FB8. Black boxes indicated observation from the proximal, middle and distal regions of inner surface of petals. The lower panel showed scanning electron microscope images of granule-shape wax structure on the surface of epidermal cells at 2000x, 4000x and 1200x magnifications. **(C)** Purple pigmentation of corolla tube. The upper panel showed the photos of purple pigmentations on the abaxial side of dorsal petal and the adaxial side of ventral petal at stages of FB5, FB8 and FB16. The lower panel summarized the pigmenting patterning of epidermis on dorsiventral petals at stages of FB3, FB5, FB8 and FB16 by cartoon illustration.

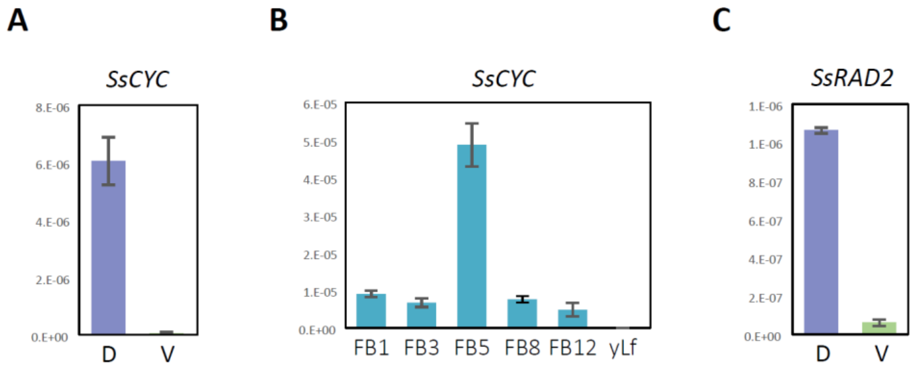

Supplementary Figure S2. Expression patterns of the floral symmetry genes in *S. speciosa* 'ES'. Normalized expression levels of *SsCYC* **(A)** in the dorsal and ventral petals at FB5 stage and **(B)** in floral buds without sepals at five developmental stages and in young leaves. (**C**) *SsRAD2* in the dorsal and ventral petals at FB5 stage. D, dorsal petals; V, ventral petals; yLf, young leaf.

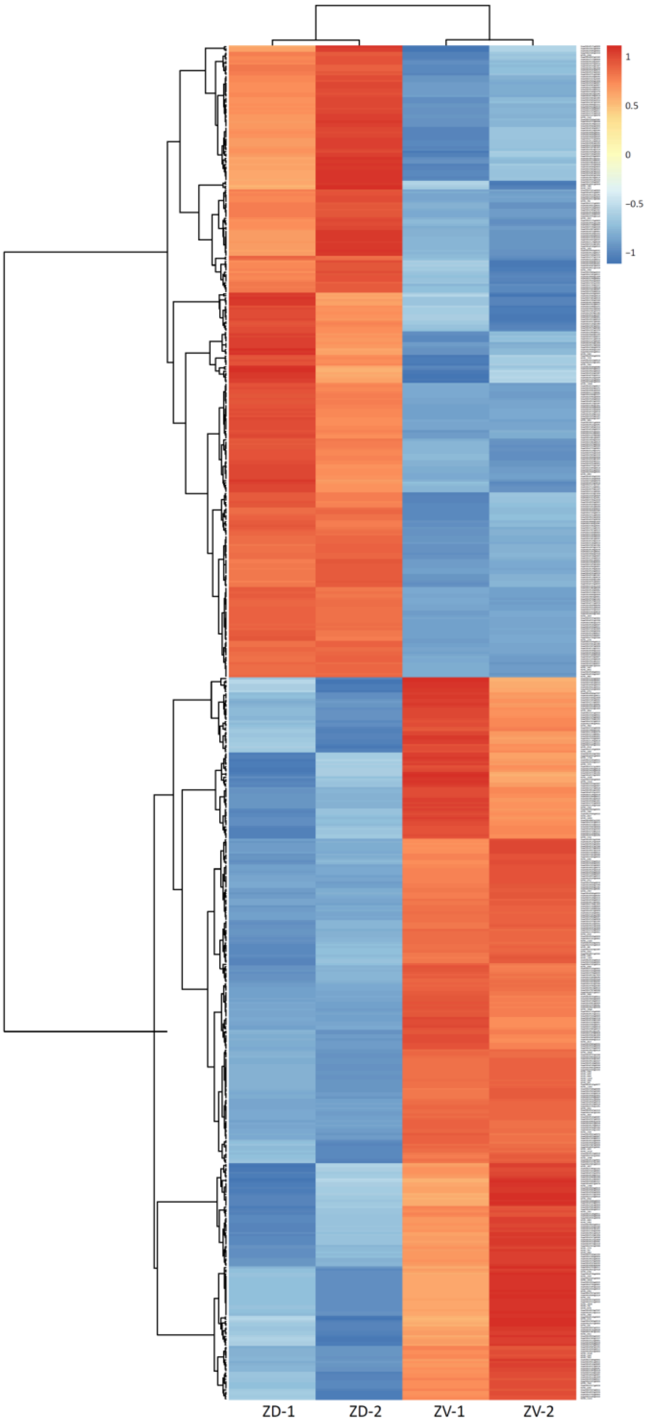

Supplementary Figure S3. Hierarchical cluster analysis of the expression patterns of DV-DEGs in the ZD-1, ZD-2, ZV-1, and ZV-2 RNA-seq libraries. The relative expression levels of each genes (rows) in each samples (columns). Normalized TMM expression values are log2-transformed. The expression values are plotted in red-blue color scale with red and blue indicating increased and decreased expressions, respectively.

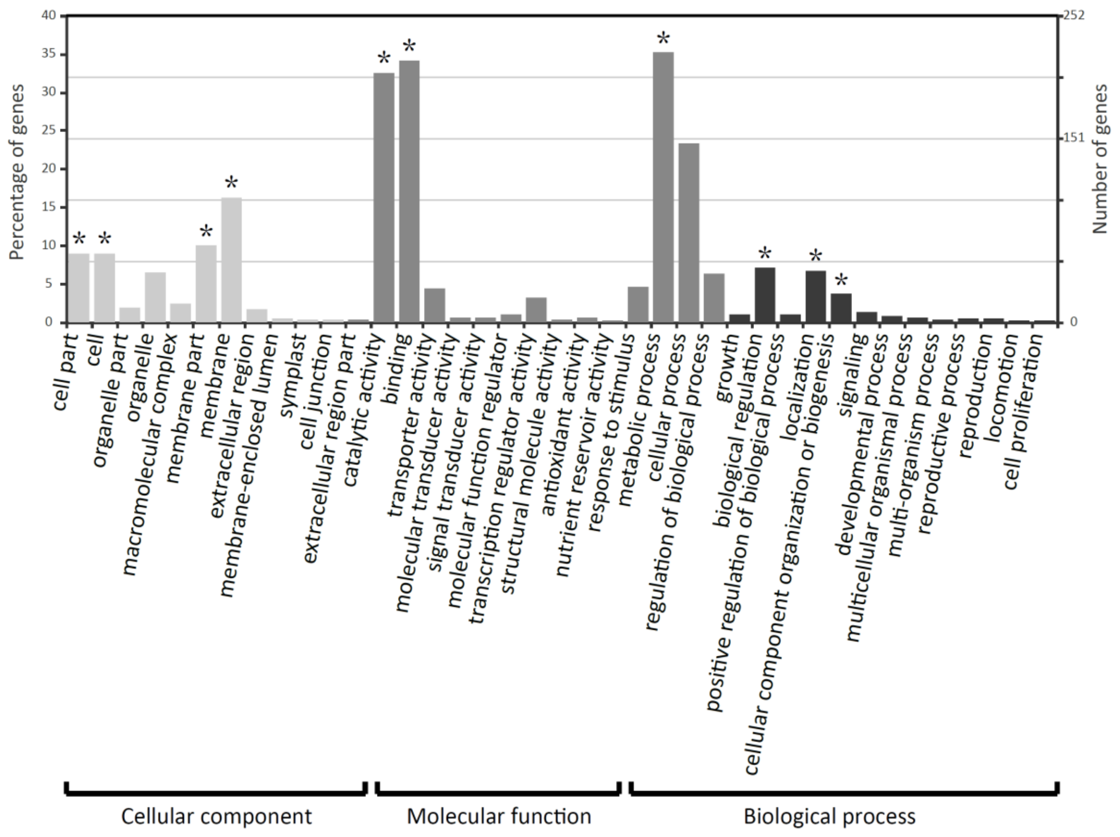

Supplementary Figure S4. Gene Ontology (GO) annotation of DV-630 DEGs from dorsiventral petal transcriptome. Annotated hits were classified under three main GO categories: cellular component, molecular function and biological process. The star marked the top 3 highly abundant subcategories in each main categories. The left and right y-axis indicated the percentage and number of a specific subcategory.

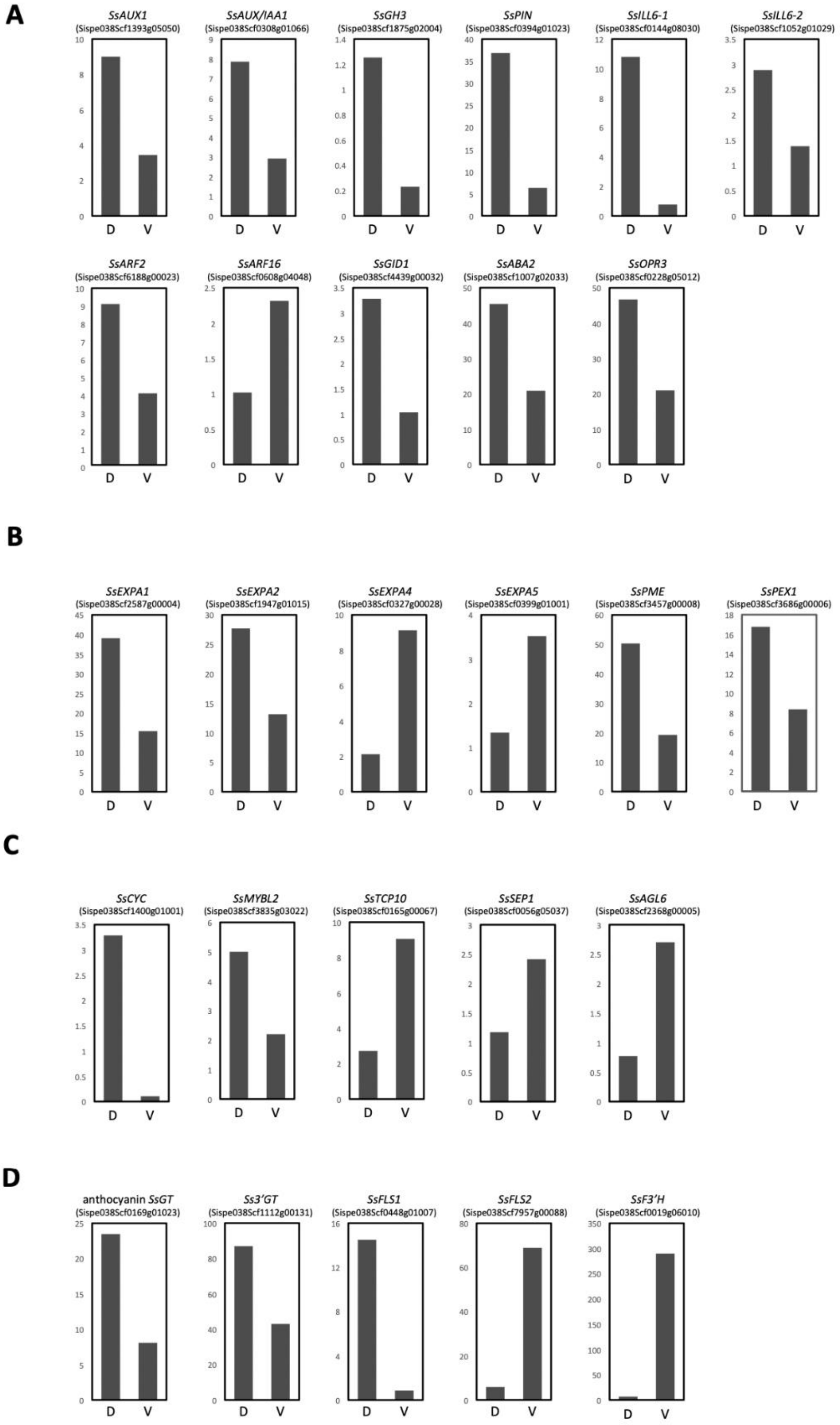

Supplementary Figure S5. Transcriptional levels of DEGs from the determined RNA-Seq analysis. Annotated DEGs involved in processes of **(A)** auxin and other hormone pathways; **(B)** cell wall modification; **(C)** transcription factors and **(D)** flavonoid biosynthesis pathway. D, dorsal petals; V, ventral petals.

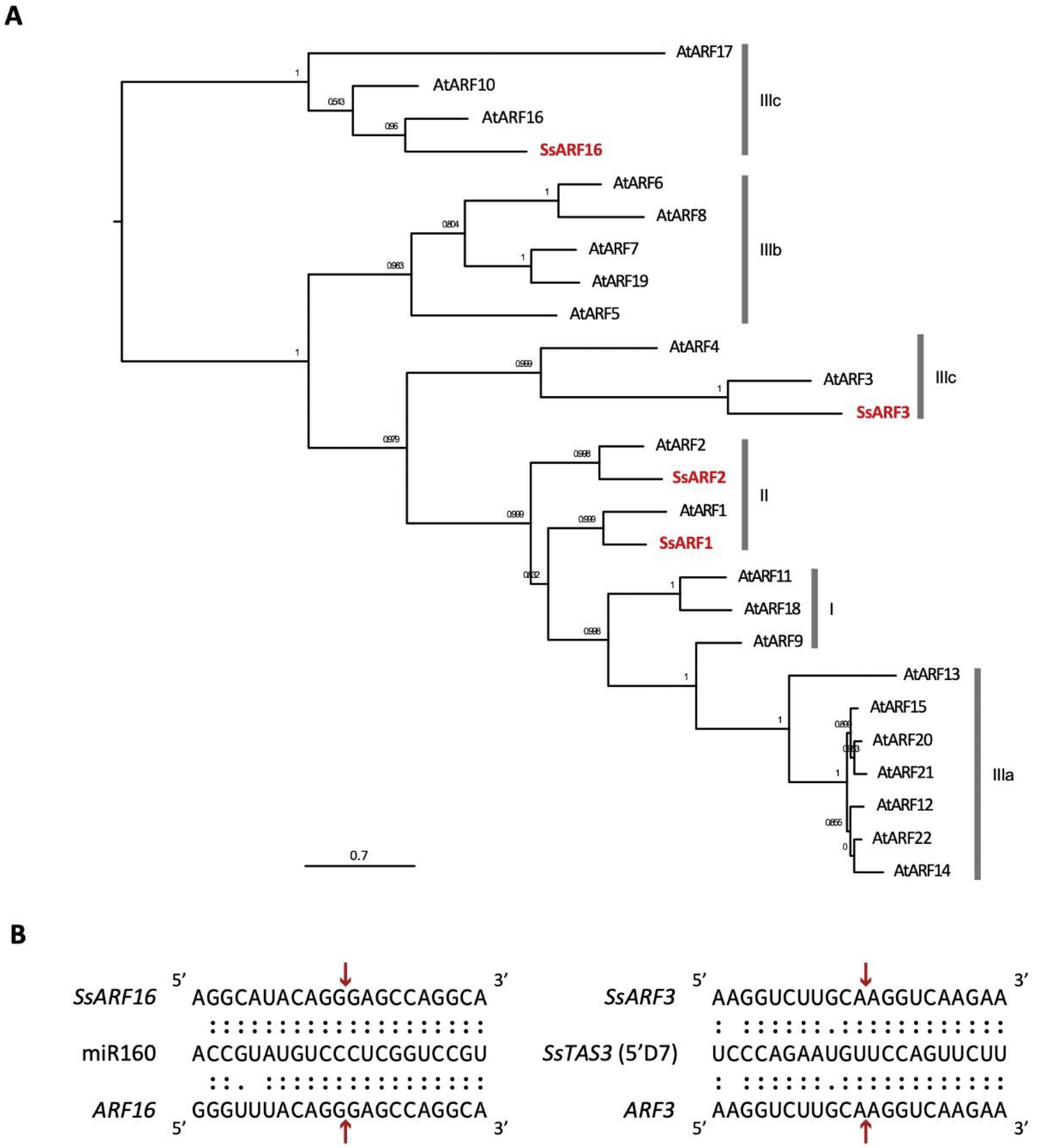

Supplementary Figure S6. Sequence analysis of *SsARFs* of *S. speciosa* 'ES'. **(A)** ML phylogenetic tree of *ARF* homologs from *A. thaliana* and *S. speciosa* 'ES'. The bootstrap values are denoted above the nodes. The *SsARFs* were marked in bold and only the differential expressed *SsARF16* were marked in red. **(B)** Sequence pairing between miR160 and *ARF16* and *SsARF16* and between *TAS3* ta-siARF and *ARF3* and *SsARF3*. The predicted cleavage sites on targets are marked by red arrows.

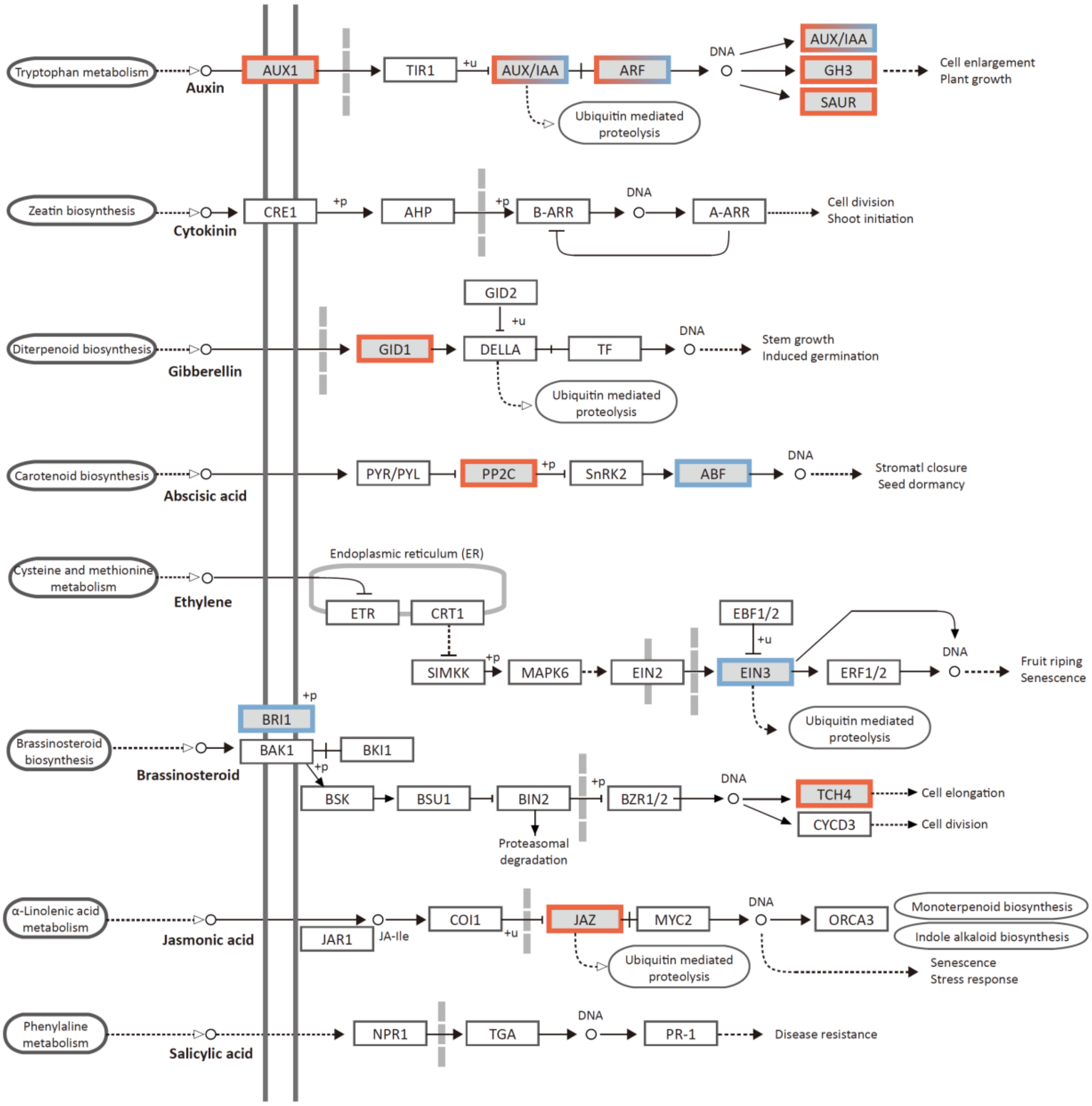

Supplementary Figure S7. DEGs assigned to the eight plant hormone signal transduction pathways. Grey boxes indicated pathway genes mapped by DEGs. Outline colors in red, blue and black represented dorsal-high, ventral-high and no differentially expressed genes.

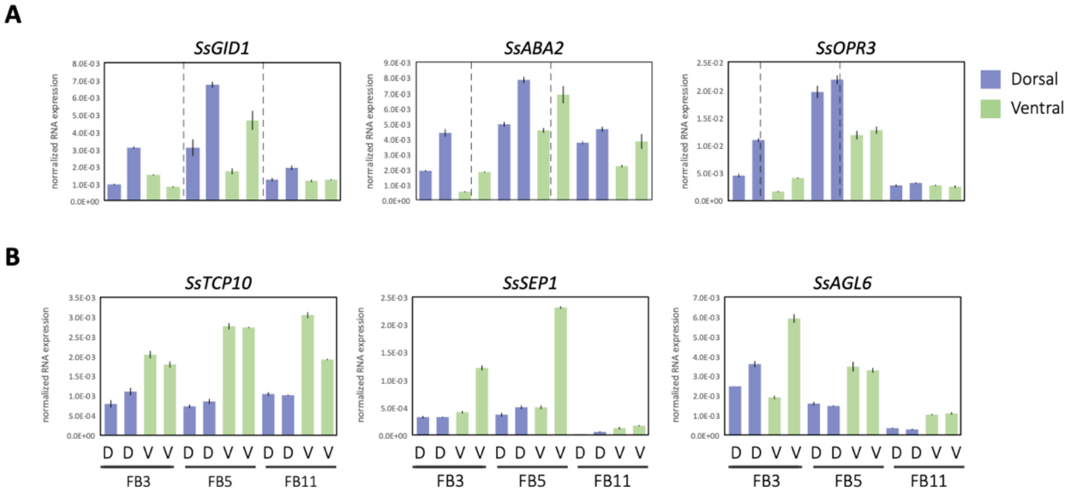

Supplementary Figure S8. Validation of expression patterns of DEGs in the dorsal and ventral petals at developmental stages FB3, FB5, and FB11 in *S. speciosa* 'ES' flowers by qRT-PCR. **(A)** Annotated DEGs, *SsGID1*, *SsABA2* and *SsOPR3* involved in the processes of GA, ABA and JA pathways, respectively. **(B)** *SsTCP10*, *SsSEP1* and *SsAGL6* were transcription factor genes. D, dorsal petals; V, ventral petals.

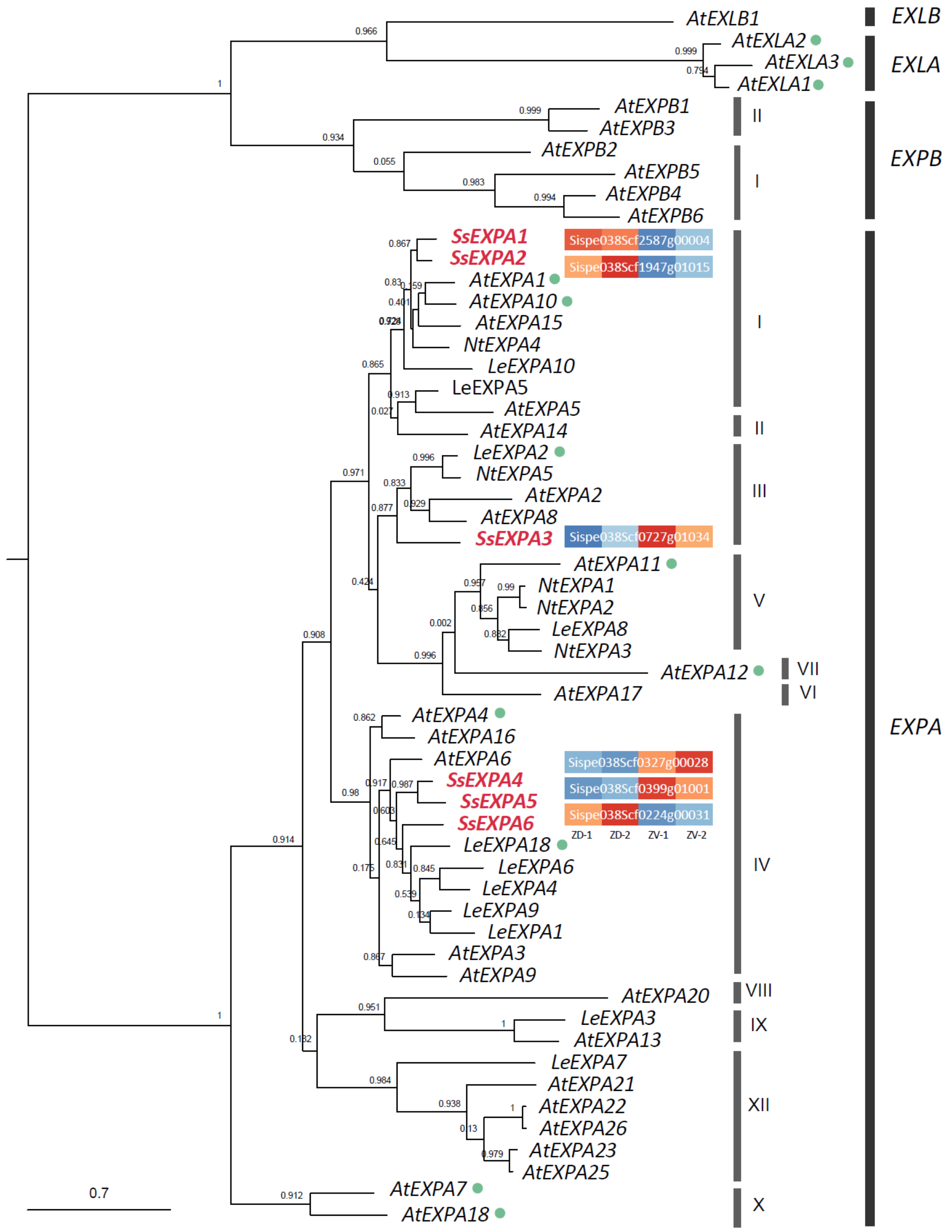

Supplementary Figure S9. ML phylogenetic tree of the *EXPANSIN* gene family. The sequences of *AtEXPs* (*A. thaliana*), *LeEXPs* (*Solanum lycopersicum*), *NtEXPs* (*N. tabacum*), and *SsEXPs* (*S. speciosa* 'ES') were used to construct the ML tree. The bootstrap values are shown above the branches at the nodes. The *SsEXP* genes from *S. speciosa* are shown in red. Heatmaps on the right side show the relative expression levels of *SsEXP* genes in the dorsal and ventral petals determined from RNA-seq data. Green circles indicate previous reported that *EXP* homologous genes in which expression is induced by treatment with IAA or picloram (Majda and Robert, 2018).

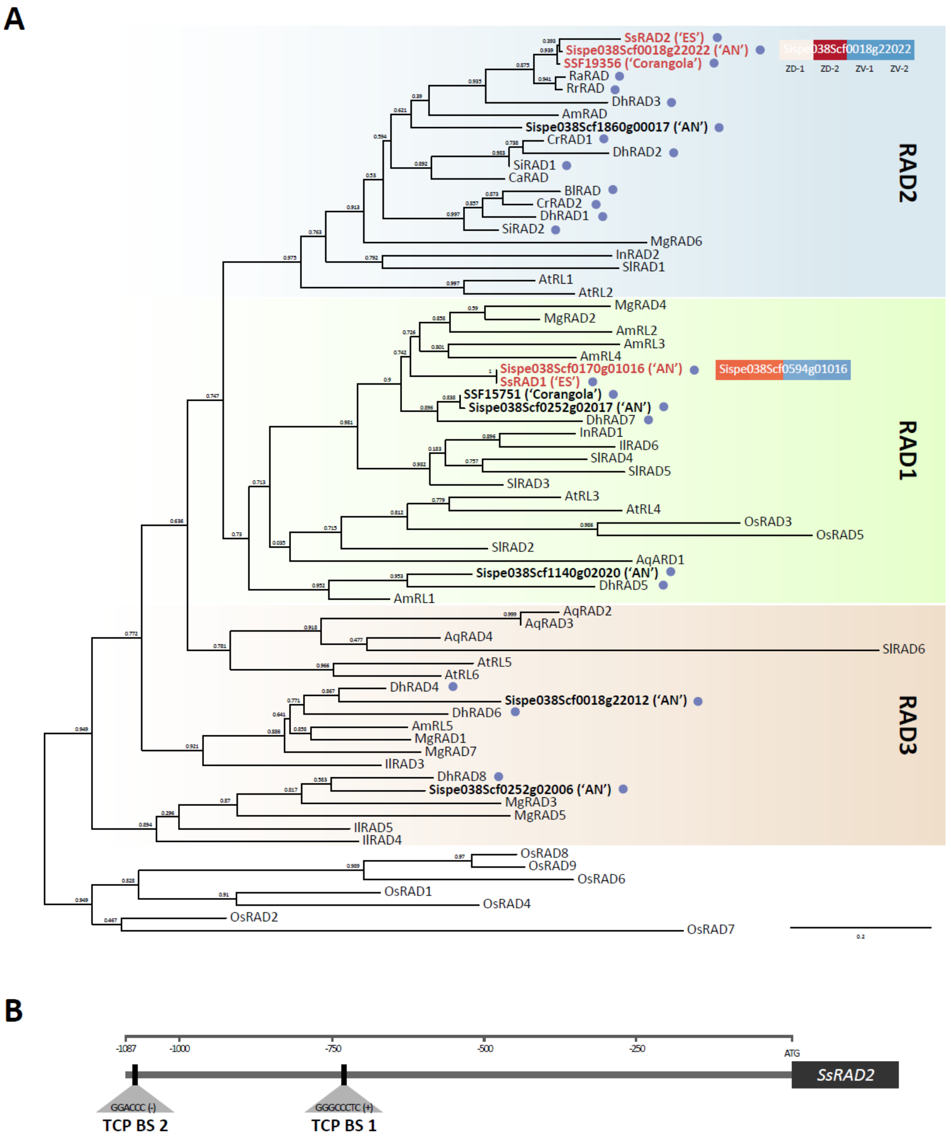

Supplementary Figure S10. Sequence analysis of *SsRAD* genes of *S. speciosa* 'ES'. **(A)** ML phylogenetic tree of *RADIALIS*-like genes. The bootstrap values are denoted above the nodes. The *RAD* homologs from *S. speciosa* of three cultivars (Sispe, 'Avenida Niemeyer'; SSF, 'Corangola' F2; ES, 'Espirito Santo') were marked in bold and only the differential expressed *SsRADs* were marked in red. Purple circles represented genes belong to Gesneriaceae family. Homologs of RAD from *Antirrhinum majus* (Am), *Arabidopsis thaliana* (At), *Aquilegia coerulea* (Aq), *Bournea leiophylla* (Bl), *Callicarpa americana* (Ca), *Conandron ramondioides* (Cr), *Dorcoceras hygrometricum* cultivar XS01 (Dh), *Ipomoea nil* (Il, In), *Mimulus guttatus* (Mg), *Oryza sativa* (Os), *Streptocarpus ionanthus* (Si), *Solanum lycopersicum* (Sl), *Rhytidophyllum auriculatum* (Ra) and *Rhytidophyllum rupincola* (Rr) **(B)** The TCP binding sites (TCP BSs) predicted on the 5'region of *SsRAD2*.

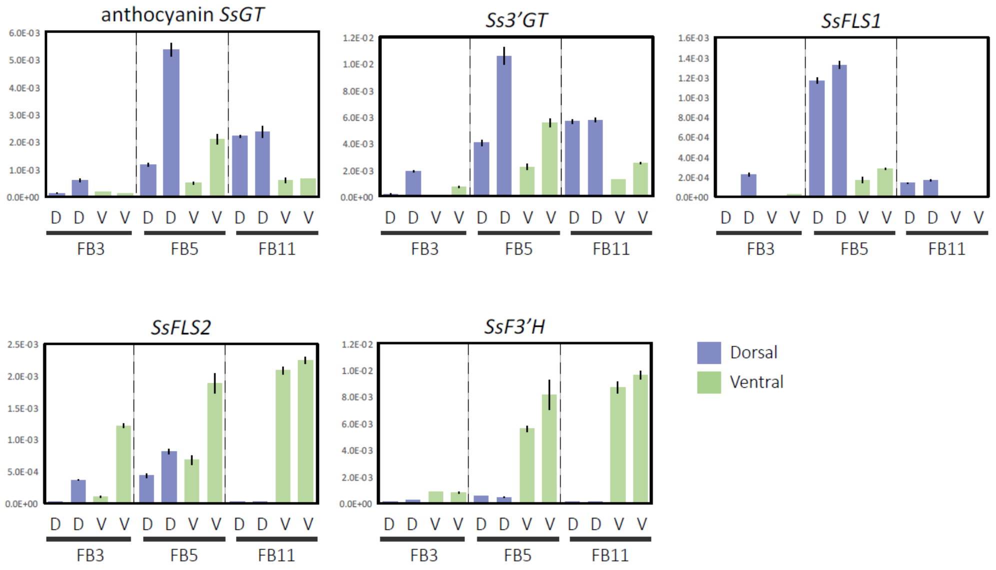

Supplementary Figure S11. Validation of expression patterns of DEGs in the dorsal and ventral petals at developmental stages FB3, FB5, and FB11 in *S. speciosa* 'ES' flowers by qRT-PCR. Annotated DEGs, *SsFLSs* (flavonol synthase), *SsF3'H* (flavanoid 3'-hydroxylase) and *SsGTs* (UDP-glucose: flavonoid 3-O-glucosyltransferase). D, dorsal petals; V, ventral petals.

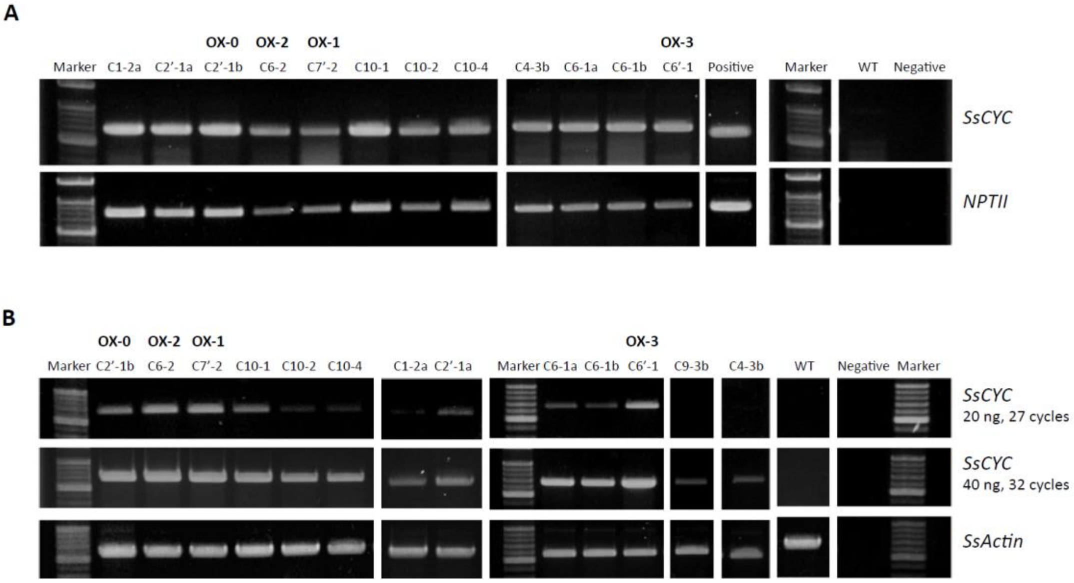

Supplementary Figure S12. T-DNA insertion and mRNA expression of *p35S:SsCYC-c-Myc* T_0 transgenic plants. **(A)** PCR of *SsCYC* and *NPTII* gene insertions. **(B)** RT-PCR of *SsCYC* gene expression levels. *SsActin* as the house keeping gene. Wild type, WT.

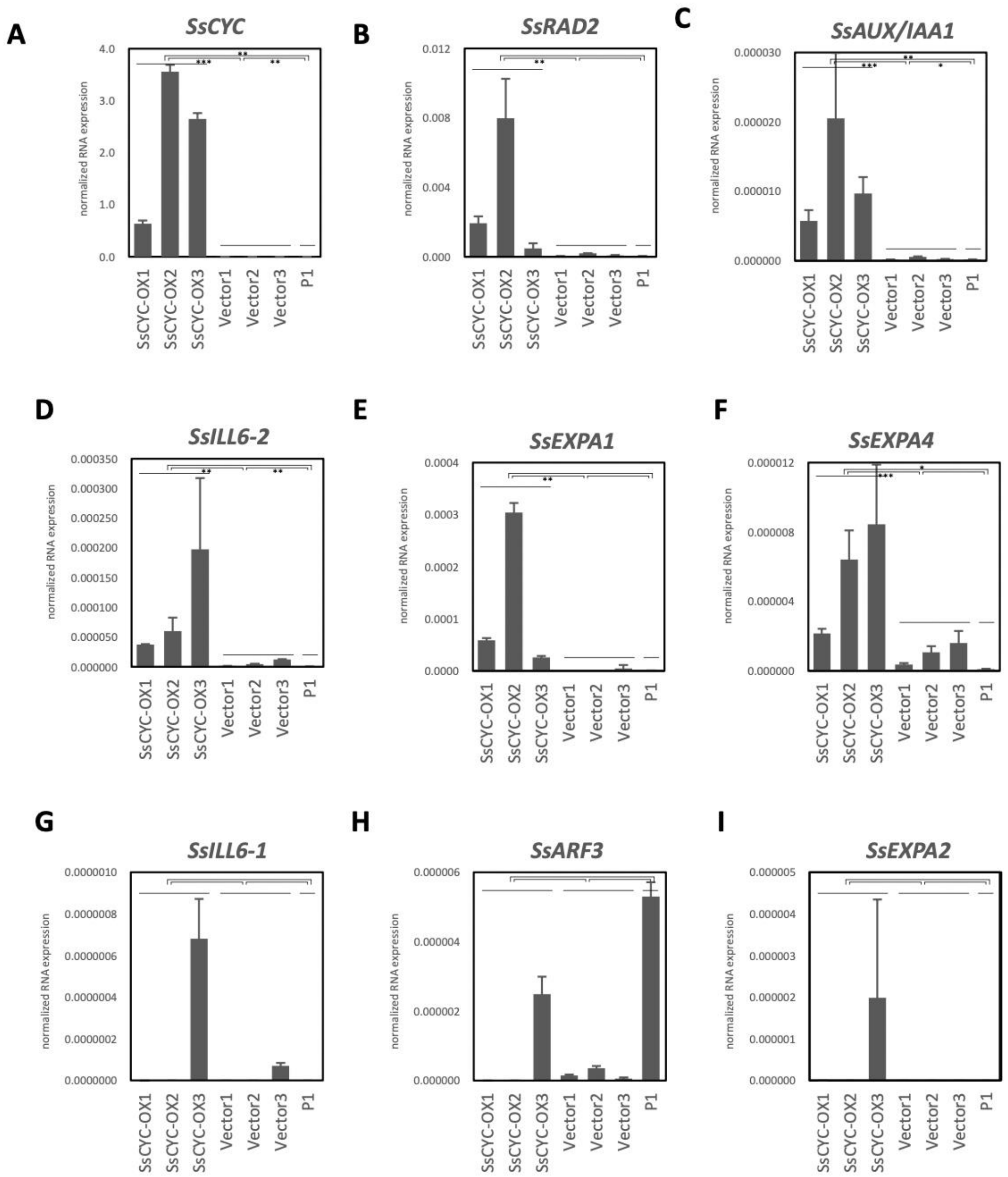

Figure S13. Induced expressions of DV-DEGs by *SsCYC* in petal protoplasts of *S. speciosa*. The normalized expression levels of *SsCYC*-*SsRAD* among three transfection lines were indicated in **(A-B)**. The induced expression levels of DEGs in the auxin pathway **(C-D, G-H)** and cell wall modification **(E-F, I)** of these *SsCYC* overexpression lines were compared. For each gene, the expression levels of protoplasts transfected with effector vector containing *35S:SsCYC* (OX) were compared to those with the empty vector (Vector) or to un-transfected protoplasts for negative controls (P1). The mean values ± *SD* are from three technical repeats. Statistical analysis was determined by Student's *t* test, **p* < 0.05, ***p* < 0.01, ****p* < 0.001.
